# Face dissimilarity judgments are predicted by representational distance in morphable and image-computable models

**DOI:** 10.1073/pnas.2115047119

**Published:** 2022-06-29

**Authors:** Kamila M. Jozwik, Jonathan O’Keeffe, Katherine R. Storrs, Wenxuan Guo, Tal Golan, Nikolaus Kriegeskorte

**Affiliations:** ^a^Department of Psychology, University of Cambridge, Cambridge CB2 3EB, United Kingdom;; ^b^MRC Cognition and Brain Sciences Unit, University of Cambridge, Cambridge CB2 7EF, United Kingdom;; ^c^Department of Experimental Psychology, Justus Liebig University, 35394 Giessen, Germany;; ^d^Centre for Mind, Brain and Behaviour, University of Marburg, Justus Liebig University Giessen, 35394 Giessen, Germany;; ^e^Zuckerman Mind Brain Behavior Institute, Columbia University, New York, NY 10027;; ^f^Department of Psychology, Columbia University, New York, NY 10027;; ^g^Department of Neuroscience, Columbia University, New York, NY 10027;; ^h^Department of Electrical Engineering, Columbia University, New York, NY 10027

**Keywords:** face perception, face similarity, face identification, Basel face model, deep neural networks

## Abstract

Human vision is attuned to the subtle differences between individual faces. Yet we lack a quantitative way of predicting how similar two face images look and whether they appear to show the same person. Principal component–based three-dimensional (3D) morphable models are widely used to generate stimuli in face perception research. These models capture the distribution of real human faces in terms of dimensions of physical shape and texture. How well does a “face space” based on these dimensions capture the similarity relationships humans perceive among faces? To answer this, we designed a behavioral task to collect dissimilarity and same/different identity judgments for 232 pairs of realistic faces. Stimuli sampled geometric relationships in a face space derived from principal components of 3D shape and texture (Basel face model [BFM]). We then compared a wide range of models in their ability to predict the data, including the BFM from which faces were generated, an active appearance model derived from face photographs, and image-computable models of visual perception. Euclidean distance in the BFM explained both dissimilarity and identity judgments surprisingly well. In a comparison against 16 diverse models, BFM distance was competitive with representational distances in state-of-the-art deep neural networks (DNNs), including novel DNNs trained on BFM synthetic identities or BFM latents. Models capturing the distribution of face shape and texture across individuals are not only useful tools for stimulus generation. They also capture important information about how faces are perceived, suggesting that human face representations are tuned to the statistical distribution of faces.

## Significance

Discerning the subtle differences between individuals’ faces is crucial for social functioning. It requires us not only to solve general challenges of object recognition (e.g., invariant recognition over changes in view or lighting) but also to be attuned to the specific ways in which face structure varies. Three-dimensional morphable models based on principal component analyses of real faces provide descriptions of statistical differences between faces, as well as tools to generate novel faces. We rendered large sets of realistic face pairs from such a model and collected similarity and same/different identity judgments. The statistical model predicted human perception as well as state-of-the-art image-computable neural networks. Results underscore the statistical tuning of face encoding.

Recognizing people by their faces is a perceptual ability that is central to human social behavior (1). Despite much work on the neural and behavioral signatures of face perception (e.g., refs. 2–5), there is currently no quantitative model to predict how alike two faces will look to human observers. Advances in deep learning have yielded powerful artificial systems for face and object recognition (6–8), and three-dimensional (3D) modeling and rendering techniques make it possible to systematically explore the space of possible faces (9–11). Here we asked subjects to judge the dissimilarity of realistic face pairs generated with a morphable human-face graphics model (10). We investigate how well the statistically derived latent space of this face-generative model predicts the perceived dissimilarity among faces, compared to a wide range of alternative models.

Since faces of different people are structurally highly similar and vary along continuous dimensions (nose length, jaw width, etc.), it is helpful to think of faces as forming a continuous “face space” (12–14). A face space is an abstract space in which each face occupies a unique position, and the dimensions span the ways in which physiognomic features can vary between faces. The origin of the multidimensional space is often defined as the average face: the central tendency of the population of all faces or, for an individual observer, the sample of faces encountered so far. There is perceptual and neural evidence that our brain’s face encoding adapts to better distinguish among the faces we encounter more frequently, both over our lifetime and within our recent experience (3, 15–17). There are many ways a computational model might represent the space of possible faces. For example, deep neural networks (DNNs) trained on face recognition represent individual faces in terms of combinations of complex nonlinear image features (e.g., refs. 18–20). By contrast, 3D morphable models represent faces in terms of a geometric mesh defining the face’s shape and a texture map defining the coloration at each point on the face (9–11, 21). Three-dimensional morphable models are useful tools in face perception research because they can be used to generate novel realistic faces for which ground-truth properties are known, which can be rendered under arbitrary viewing conditions (10, 11, 16, 22, 23).

**Fig. 1.**
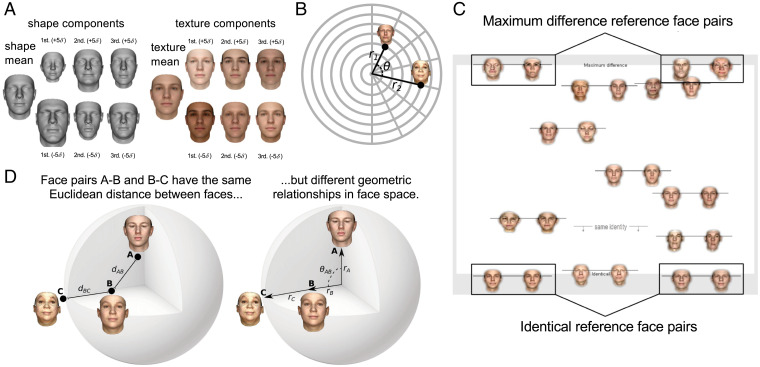
Selecting pairs of faces from the BFM, and measuring perceived face dissimilarity and identity. (*A*) Illustration of the generative BFM, in which faces are described by separate components specifying their 3D shape (*Left*) and texture (*Right*). Both shape and texture components have a mean shape or texture, and faces with diverse shape and texture appearances can be created by manipulating each set of principal components. The first three PCs within each subspace are shown here. The values of *σ* (±5*σ*) indicate moving a number of SDs away from the mean face in the direction of a given PC. Reproduced with permission from ref. 10. (*B*) Stimulus selection. We defined stimuli as pairs of vectors in the BFM with radial lengths *r*_1_ and *r*_2_ and angle between them (*θ*). We sampled all unique combinations from eight *θ* values and eight radius values to obtain 232 face pairs. See *Materials and Methods* for details. (*C*) Behavioral experiment task. Participants positioned the face pairs along the vertical axis of the screen according to their relative dissimilarity. Faces were arranged in random subsets of eight pairs, as in the example shown. The vertical position of the line linking face pairs determined their precise location. As points of reference for participants, two example face pairs depicting “maximum difference” (*Top*) and two example face pairs depicting “identical” (*Bottom*) were shown. For each subset, participants also placed a “same identity” line indicating the point below which pairs of images appear to depict the same identity. (*D*) Relationships between pairs of faces in a face space like the BFM can be thought of in terms of the Euclidean distance between them (*Left*) or their geometric relationships relative to the center of the face space (*Right*). If perceived dissimilarity can be predicted from the Euclidean distance alone, then face pair AB should look exactly as similar to one another as face pair BC. However, if observers take the angular and radial geometry of face space into account, they may have substantially different similarities.

We used the Basel face model (BFM) (9, 10), a widely used 3D morphable model in both computer graphics and face perception research (e.g., refs. 24, 25). The BFM is a 3D generative graphics model that produces nearly photorealistic face images from latent vectors describing shape and texture of the surfaces of natural faces (Fig. 1*A*). The model is based on principal component analysis (PCA) of 3D scans (acquired using a coded light system) of 200 adult faces (10). We used the BFM both as a stimulus generator and as a candidate model of face representation to explain human face dissimilarity judgments. One appealing aspect of 3D morphable models like the BFM is that they provide a reference face space, enabling researchers to systematically vary the similarity of experimental faces. However, distances within the BFM are defined in units of SD within the sample of scanned faces that the model is based on. Distances, thus, are measured relative to the variance of facial features across real individuals. It is an empirical question with no obvious answer whether distances within such a statistical face space predict perceived dissimilarities.

The goal of our study was to better understand the human perceptual face space by comparing it to diverse candidate model face spaces. We are particularly interested in how perceived similarity relates to the statistically defined face space of the BFM model. Is perception sensitive to the statistical distribution of face variation captured in this model, or is perceived similarity dominated by simpler considerations like how similar two faces are as 3D geometries or two-dimensional (2D) images? The idea that face perception is “statistically tuned” has long been suggested, in the neurophysiological (16) and psychological literature (12). Our paper reports a comprehensive test of a statistical face-space model of human face similarity percepts, by measuring how well distances in face space predict a rich dataset of human dissimilarity and identity judgments. We compare the statistical face-space model to a large set of alternative metrics and models, which enables us to evaluate a wider range of hypotheses. Only 4 of the 16 models (pixel, VGG-Object, VGG-Face, and active appearance model) were considered in previous works (22, 23, 26). The 16 models comprise low- and midlevel image descriptors (pixel, GIST), a 2D Eigenface model, a 2D-morphable active appearance model (27, 28), a 3D-mesh model capturing the shape of a face, three facial configural models, the angle about the average face in BFM face space, the full BFM model capturing shape and texture, and six deep neural network models with different architectures (VGG, Alexnet, HMAX) and training objectives (face-identity recognition, face-latent prediction, object classification).

**Fig. 2.**
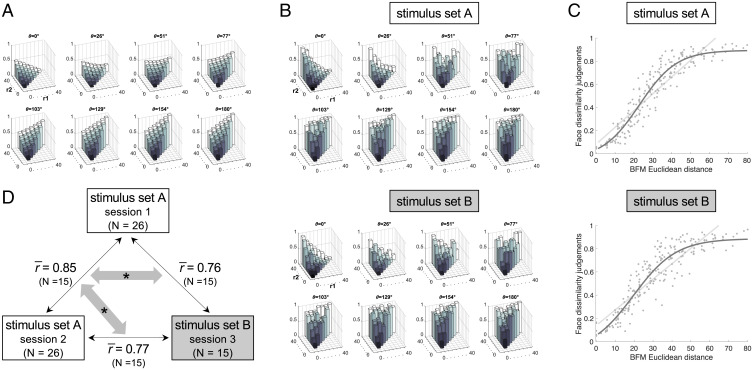
Face dissimilarity judgments as a function of distances in the BFM. (*A*) Euclidean distances within the BFM for each face pair in the stimulus sets. Sets A and B had identical BFM geometries, but used different specific face exemplars. Each plot shows pairs of face vectors separated by a specific angle (*θ*) and arranged by the lengths of each of the two vectors (*r*_1_ and *r*_2_). Radii were sampled in eight evenly spaced steps, from a length of zero (black) to a length of 40 units in the BFM (white). Normalized Euclidean distance between faces in each pair is indicated by the height of each bar, from zero (identical) to one (maximum distance within the stimulus set). (*B*) Human face dissimilarity judgments for each face pair, for the stimulus sets A (*Top*) and B (*Bottom*). Plotting conventions are as in *A*, except that bar height indicates human-rated dissimilarity, from zero (identical faces) to one (maximally dissimilar faces), averaged over participants and trials. (*C*) Face dissimilarity judgments (*y* axis) as a function of Euclidean distance in the BFM (*x* axis) for the stimulus sets A (*Top*) and B (*Bottom*). Each dot represents the mean dissimilarity rating for one pair of faces, averaged across participants and trials. The light gray line represents the fit of a linear function and the dark gray line represents the fit of a sigmoidal function to the data. (*D*) Replicability of face dissimilarity judgments between sessions 1 and 2 (using stimulus set A and the same participants) and session 3 (using stimulus set B and a subset of the participant group). All $\overset{\text{-}}{r}$ values are averages of between-session Pearson correlation coefficients across the subset of 15 participants that participated in all three sessions. Gray arrows with asterisks indicate significantly different correlations (one-tailed paired *t* test across the 15 participants, *P* = 0.00000004; one-tailed paired Wilcoxon signed-rank test, *P* = 0.00003). The correlation of face dissimilarity judgments between the two sessions using stimulus set A (sessions 1 and 2) is higher than the correlation of judgments between stimulus sets (sessions 1 and 3 or sessions 2 and 3) for each of the 15 participants.

To efficiently acquire high-fidelity dissimilarity and identity judgments, we developed a face-pair arrangement task, in which subjects dragged and dropped face pairs on a large touchscreen in the laboratory (29). We generated face pairs to systematically sample geometric relations in statistical face space, exhaustively sampling all combinations of facial vector lengths and angles (Figs. 1*B* and 2*A* and *Materials and Methods*). This experimental design enabled us to test how well distances and angles in the BFM’s statistical space predict human dissimilarity judgments. The BFM graphics model enables us to investigate the perceptual metric of face similarity more systematically than face photographs would. We trade photorealism and real-world variability for a densely, systematically sampled face space where distances correspond to statistical variations in facial shape and texture. The 3D face mesh coordinates of BFM also enable us to test derived model representations, including the 3D-mesh shape model, the three configural models, and the BFM angle model. Finally, we are able to assess the isotropy and uniformity of perceptual dissimilarities relative to the BFM reference face space.

During the task, participants arranged pairs of face images on a large (96.9 × 56 cm) touchscreen according to how similar they appeared, relative to anchoring face pairs at the top (maximally different, diametrically opposed poles in BFM space) and bottom (identical) of the screen and relative to other adjusted pairs (Fig. 1*C*). This task has two advantages over standard pairwise dissimilarity ratings: 1) It provides a more fine-grained continuous measure of face dissimilarity within each pair (the vertical position at which the pair was placed on the screen). 2) The judgments are anchored not just to the extreme anchor pairs provided above and below the sorting arena, but also to the other adjustable pairs within each trial. Previous studies focused either on facial similarity or on facial identity; by measuring both of them in the same task we are able to evaluate categorical same/different identity judgments in the context of continuous dissimilarity judgments. We also tested whether the relative geometry within BFM was perceptually isotropic. Therefore, the stimulus set A and stimulus set B experiments had the same relative geometries but different face exemplars (Fig. 1*D*). We investigated both the continuous aspect of human face perception (graded dissimilarity) and its categorical aspect (same/different identity) by having participants place an identity threshold line in each trial’s arrangement (Fig. 1*C*). Each subject arranged the total of 232 face pairs, across 29 trials, each of which contained a random partition of 8 face pairs from the total set of pairs.

## Results

Participants (*n* = 26) were highly reliable in their dissimilarity judgments using the arrangement task (mean correlation between participants = 0.80, mean correlation for the same participant between sessions = 0.85, the stimulus set A experiment). This provided a high-quality dataset with which to adjudicate among candidate models. We repeated the same experiment with a subset of the same participants (*n* = 15) 6 mo later, with a new independently sampled face set fulfilling the same geometric relations as the original stimulus set (stimulus set B experiment; *Materials and Methods*). Participants in the stimulus set B experiment were also highly reliable in their dissimilarity judgments (mean correlation between participants = 0.79). This level of replicability allowed us to evaluate to what extent dissimilarity judgments depend on idiosyncrasies of individual faces and to what extent they can be predicted from geometric relations within a statistical face space.

### Face Dissimilarity Judgments Are Well Predicted by Distance in BFM Face Space.

We first asked how well human face dissimilarity judgments could be predicted by distances within the BFM, the principal component–based face space from which our stimuli had been generated. Since we had selected face pairs to exhaustively sample different geometric relationships within the BFM, defined in terms of the angle between faces and the radial distance of each face from the origin, we were able to visualize human dissimilarity ratings in terms of these geometric features (Fig. 2*B*). The patterns of human dissimilarity ratings closely resembled the patterns of Euclidean distances among our stimuli in the BFM space (Fig. 2*A*). Given this, we plotted dissimilarity judgments for each face pair as a function of the Euclidean distance in the BFM (Fig. 2*C*). To quantify how well the BFM approximates face dissimilarity judgments, we tested which functions best capture the relationship between behavioral dissimilarity judgments and BFM distances. We plotted the predictions of each fitted function over the data and compared their goodness of fit. If the BFM is a perfect approximator of face dissimilarity judgments, a linear function would best describe the relationship between face dissimilarity judgments and the Euclidean distances in the BFM. We do not find this assumption to be completely true as the sigmoidal function better describes the relationship between face dissimilarity judgments and the Euclidean distances in the BFM (Fig. 2*C*; the goodness of fit of linear function = 0.82, the goodness of fit of sigmoidal function = 0.86, one-sided Wilcoxon signed-rank test, P<0.05). The sigmoidal relationship between the BFM and perceived distances suggests that observers have maximal sensitivity to differences between faces occupying moderately distant points in the statistical face space, at the expense of failing to differentiate between different levels of dissimilarity among very nearby or very far apart faces. This latter result may be related to the fact that faces with very large Euclidean distances in the BFM look slightly caricatured to humans (*SI Appendix*, Figs. S1–S3). We observed similar results in the stimulus set B experiment (face dissimilarity judgments using different face pairs with the same geometrical properties as in the stimulus set A experiment; see *Materials and Methods* for details and Fig. 2*C*). This result suggests that the sigmoidal relationship between the BFM and perceived distances is observed regardless of the face pairs sampled. Overall, the BFM is a good, but not perfect, approximator of face dissimilarity judgments.

### Face Identity Judgments Are Well Predicted by Distance in BFM Face Space.

**Fig. 3.**
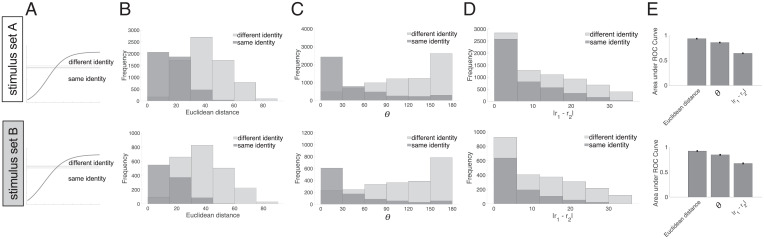
Identity judgments as a function of geometry within the BFM. (*A*) Threshold for judging faces as belonging to the same/different identity, visualized relative to similarity judgments. The curved line shows the sigmoidal fit to face dissimilarity judgments (from Fig. 2*C*) in the stimulus sets A (*Top*) and B (*Bottom*). The thick horizontal line shows the mean placement of the “different identity” threshold line, across participants and trials; thinner lines above and below indicate the SEM over participants. (*B*) Histogram of how frequently face pairs were judged as having the same identity (dark gray) or different identity (light gray), as a function of their Euclidean distance in the BFM. (*C*) Histogram of same and different identity judgments as a function of angle (*θ*) between faces in the BFM. (*D*) Histogram of same and different identity judgments as a function of the absolute difference between vector lengths in the BFM (*r*_1_ and *r*_2_). (*E*) Summary of how well each of the three BFM metrics in *B–D* discriminates face pairs judged as having the same vs. different identity. Bars show the area under the ROC curve calculated based on identity judgments using Euclidean distance, *θ*, and the absolute difference between *r*_1_ and *r*_2_.

We also asked humans to judge whether each pair of faces depicted the same or different identity and examined human identity thresholds in relation to the Euclidean distance between faces in the BFM. We found that moderately dissimilar faces in terms of the BFM Euclidean distance are often still perceived as having the same identity (Fig. 3*A*). We observed similar results in the stimulus set B experiment (Fig. 3*A*). This result may be related to humans having a high tolerance to changes in personal appearance due to age, weight fluctuations, or skin complexion depending on the season.

Face pairs in the BFM can be analyzed in terms of their geometric characteristics relative to the center of the face space or as the Euclidean distance between them. Therefore, we tested alternative predictors of face identity judgments: geometry in the BFM (*θ*, absolute difference between *r*_1_ and *r*_2_) and the Euclidean distance. We could predict whether two faces will be classified as the same individual by each of the predictors (Fig. 3 *B*–*D*). The Euclidean distance in the BFM predicted identity judgments marginally better than the angular and radial geometry of face space (Fig. 3*E*).

### Dissimilarity Judgments Are Approximately Isotropic in BFM Face Space.

A representational space is perceptually isotropic if perceived dissimilarity remains constant as the direction of the pair of face vectors is rotated in any direction around the origin (while preserving their lengths and angle; Fig. 1*D*). We cannot test for isotropy in the stimulus set A experiment, because for each geometric relationship [(*θ*), r1, r2] we have only one sample of face pairs. To address this limitation, we compared the responses to the stimulus set A with either a repeated measurement of the responses to the same stimulus set or the responses to stimulus set B, which had the same relative geometries but different face exemplars (i.e., each face pair had a different direction in BFM space). Participants completed two sessions out of the stimulus set A experiment and a subset of participants (15 of 26) completed the third session of the stimulus set B experiment. If the relative geometry within the BFM is isotropic, then the correlation between stimulus set A and B experiments should be the same as the correlation between two sessions of the stimulus set A experiment. The correlation between two sessions of the stimulus set A experiment for the subset of 15 participants is 0.85, the correlation between the stimulus set A session 1 and stimulus set B session 3 experiment is 0.76, and the correlation between the stimulus set A session 2 and stimulus set B session 3 experiment is 0.77 (Fig. 2*D*). Replicability of face dissimilarity judgments between sessions 1 and 2 (using stimulus set A and the subset of 15 participants) and session 3 (using stimulus set B and a subset of the participant group) is significantly higher when the same stimulus set was used (one-tailed paired *t* test across the 15 participants, *P* = 0.00000004; one-tailed paired Wilcoxon signed-rank test, *P* = 0.00003). The correlation of face dissimilarity judgments between the two sessions using stimulus set A (sessions 1 and 2) is higher than the correlation of judgments between stimulus sets (sessions 1 and 3 or sessions 2 and 3) for each of the 15 participants. These results suggest that the relative geometry within the BFM is approximately, but not exactly, perceptually isotropic. However, since the stimulus set B experiment was performed 6 mo after the stimulus set A experiment, the decreased correlation with stimulus set B could be attributed to the longer time between sessions with different face exemplars. Hence, we do not interpret the correlation difference as strong evidence against isotropy.

A related concept to isotropy is face space uniformity. The space is perceptually uniform if perceived dissimilarity remains constant as the pair of face points is translated anywhere in the space. This is a looser requirement than isotropy because all it preserves is the Euclidean distance between face points, not their geometry relative to the origin. For example, if the space is uniform, then it should not matter whether one face is at the origin and the other is 10 units away or both faces are 5 units away from the origin in opposite directions. We searched for evidence of perceptual uniformity in the stimulus set A experiment, by binning face pairs into groups with similar Euclidean distance and then evaluating whether the angle between the faces explains variance in perceived dissimilarity. If the space is nonuniform, we might expect faces with larger angular differences to appear more different, even if they have identical Euclidean distance. We find only weak evidence for any nonuniformity in the face space (*SI Appendix*, Fig. S4).

### Deep Neural Networks Trained on Diverse Tasks Predict Perceived Face Dissimilarity Well.

**Fig. 4.**
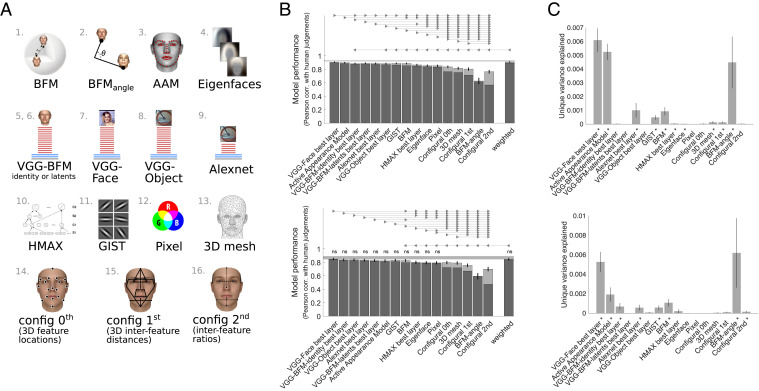
Comparing diverse models in their ability to predict face dissimilarity judgments. (*A*) Schematic illustration of models compared (see Table 1 for full details). Two models were based on the 3D morphable model from which faces were generated: 1) Euclidean distance in the 3D morphable model and 2) cosine distance within the full BFM coordinate space. Other models included 3) AAM and 4) Eigenfaces. DNN models consisted of a 16-layer VGG architecture trained on 5), 6) BFM faces, 7) face photographs, or 8) objects and 9) an 8-layer Alexnet architecture trained on objects. Alternative models were 10) a shallower HMAX neural network; 11) GIST image descriptors; 12) raw pixel values; 13) raw 3D face mesh; and configural models 14) “0th order” configuration (location of 30 key points such as eyes, nose, mouth), 15) “1st order” configuration (distances between key points), and 16) “2nd order” configuration (ratios of distances between key points). (*B*) Ability of each model to predict face dissimilarity judgments in the stimulus sets A (*Top*) and B (*Bottom*). Bars show a Pearson correlation between human-judged face dissimilarity and face-pair distance within each model. The dark lower region of each bar shows performance for raw model distances, while the lighter upper region shows additional performance gained if model distances are transformed by a compressive nonlinearity (a sigmoidal function fitted to data from training participants and face-pair stimuli). All models were significantly correlated with human data (*P*  <  0.05 corrected). The gray bar represents the noise ceiling, which indicates the expected performance of the true model given the noise in the data. The final bar shows the performance of a linear weighted combination of all models, fitted using nonnegative least squares. Fitting of sigmoidal transforms and linear reweighting was performed within the same cross-validation procedure, fitting and evaluating on separate pools of both participants and stimuli. Error bars show the SEM (95% confidence interval over 2,000 bootstrap samples). Horizontal lines show pairwise differences in model performance (*P*  <  0.05, Bonferroni corrected across all comparisons). Models connected by triangular arrow markers indicate a significant difference, following the convention in ref. 60, with the direction of the arrow marker showing which model is superior. All statistical tests shown were performed on the untransformed version of each model. For statistical comparisons among models with sigmoidal transform, see *SI Appendix*, Fig. S9. (*C*) Unique variance in face dissimilarity judgments computed using a hierarchical GLM for the stimulus sets A (*Top*) and B (*Bottom*). For each model, unique variance is computed by subtracting the total variance explained by the reduced GLM (excluding the model of interest) from the total variance explained by the full GLM, using nonnegative least squares to find optimal weights. Models that explain significant unique variance are indicated by an asterisk (one-sided Wilcoxon signed-rank test, *P*  <  0.05 corrected). Error bars show the SEM based on single-participant unique variance.

We tested a wide range of models. All models tested are schematically presented in Fig. 4*A*. We considered Euclidean or cosine distances within the full BFM space. Simple alternative models consisted of a 3D mesh model, RGB pixels, GIST, Eigenfaces, and face configurations: “zeroth-order” configuration (location of 30 key points such as eyes, nose, mouth), “first-order” configuration (distances between key points), and “second-order” configuration (ratios of distances between key points). We also considered an active appearance model (27), trained to summarize facial shape and texture of natural faces (28). Finally, the last class of models consisted of DNNs of either a 16-layer VGG (30) or an 8-layer Alexnet architecture (31). VGG was trained either to recognize objects, identify faces from real-world photographs, and recognize synthetic identities generated from the BFM (*SI Appendix*, Fig. S14) or to estimate the underlying BFM latent representation of such synthetic images (*SI Appendix*, Fig. S15). All DNNs were trained on recognition tasks or latent prediction rather than to report dissimilarity directly. Predicted dissimilarities of each face pair for each model are shown in *SI Appendix*, Fig. S5.

We inferentially compared each model’s ability to predict face dissimilarity judgments, in both their raw state and after fitting a sigmoidal transform to model-predicted dissimilarities, using a procedure cross-validated over both participants and stimuli (*Materials and Methods*). The highest-performing model was the VGG deep neural network trained on face identification (VGG-Face) (18) (Fig. 4*B*, *Top*). We included in the comparison the highest-performing layer of each deep neural network (for the comparison of all VGG layers, see *SI Appendix*, Fig. S6).To complement the neural networks trained on naturalistic images, we trained (*Materials and Methods*) the same VGG-16 architecture to classify identities (*SI Appendix*, Fig. S14) sampled from the Basel face model 2018 (32) or to estimate the underlying BFM latents of such synthetic images (*SI Appendix*, Fig. S15), an objective inspired by Yildirim et al. (23). Both of these models remove the mismatch between the training (real naturalistic faces) and test distributions (BFM) of faces. The best layer of a VGG trained on BFM identities or on BFM latents does not perform better than the best layer of a VGG trained on natural faces and does not capture any unique variance (Fig. 4*C*, *Top*). In all four VGG training scenarios (objects, natural faces, BFM faces, and BFM latents), match to human judgments peaked in late convolutional layers, before declining again in fully connected layers (for the comparison of all VGG–BFM-identity layers and VGG–BFM-latents layers, see *SI Appendix*, Figs. S7 and S8). Human face perception is captured by a DNN that has experience with real naturalistic faces; and there is no evidence that the performance of a DNN trained on real faces is diminished by testing it on generated faces. Several other models performed well, and the active appearance model (AAM) was not statistically different from VGG-Face. Euclidean distance within BFM was outperformed by VGG-Face and AAM, but not by any of the other models (Fig. 4 *B*, *Top*). Performing the same analysis on the independent stimulus set B experiment revealed good reproducibility of the model rankings, even though the stimulus faces are different (Fig. 4 *B*, *Bottom*). VGG-Face again achieved the highest performance, but in this dataset was not significantly superior to several other models: VGG–BFM-identity, VGG-Object, Alexnet, active appearance model, and GIST. Again, the BFM model was competitive with image-computable models, being outperformed only by VGG-Face and VGG–BFM-latents. Most models reached the noise ceiling in this second dataset, but this is likely because there was greater overall measurement noise, due to a smaller sample size and one rather than two experimental sessions.

Like the BFM-based models, we additionally evaluated all other models after sigmoidally transforming their raw predictions (cross-validated, fitting and testing on separate participants and stimuli). Light shaded upper portions of bars in Fig. 4*B* indicate the performance of sigmoidally fitted versions of each model, and *SI Appendix*, Fig. S9 shows the result of statistical comparisons between them. All models better predicted human responses after fitting a sigmoidal function to their raw predicted distances and produced a greater relative improvement for more poorly performing models, but did not substantially affect model rankings (Fig. 4*B*). VGG-Face predicts human judgments best, and BFM distance is competitive, being outperformed only by VGG-Face in the stimulus set A and the stimulus set B experiments (*SI Appendix*, Fig. S9). Taking raw and sigmoidally transformed performance across the two datasets into account, we found no single best model, but a set of consistently very highly performing ones. The four DNN models, the 3D morphable BFM, the AAM, and the image-statistic summary GIST model all excellently predicted face dissimilarity judgments. We observed that the average correlation between the models is 0.81 (with the highest correlation being 0.99 and the lowest correlation being 0.41; *SI Appendix*, Fig. S12). These results mean that there is a different degree of similarity of model predictions between models tested.

In *SI Appendix* analyses, we evaluate a number of additional models beyond the 16 shown in Fig. 4. These BFM-based additional models are Euclidean and cosine distances within three linear subspaces of BFM face space: 1) the BFM shape dimensions only, 2) the BFM texture dimensions only, and 3) a four-dimensional subspace consisting of the dimensions capturing most variance in the “person attributes” height, weight, age, and gender (*SI Appendix*, Fig. S10). We also assessed models based on Euclidean distance within different numbers of principal components of the full BFM space (*SI Appendix*, Fig. S11). The performances are very similar for calculations performed on the full BFM space or on either the shape or the texture subspaces using Euclidean distance or angle metrics (*SI Appendix*, Fig. S10). The model with loadings on perceptually relevant dimensions of age, gender, weight, and height explained less variance than the full model (*SI Appendix*, Fig. S10). Increasing the number of the principal components in the full model leads to a rapid increase in performance as the first 1 to 10 components are included (*SI Appendix*, Fig. S11). The BFM model with 50 principal components (as used in ref. 33) is close to the performance of the full model. It seems that much smaller subspaces than the full 199-dimensional space may be sufficient to explain the variance in facial dissimilarity judgments. However, the effects of later principal components on facial appearance are likely to be less visible at a given image resolution than those of early components.

There are substantial computational differences between the several models that all predict human perceived face dissimilarity well. Do they explain shared or unique variance in human judgments? To address this question we performed a unique variance analysis on all models. Several models explained a significant amount of unique variance, especially VGG-Face, BFM angle, and AAM models in both stimulus set A and stimulus set B experiments (Fig. 4*C*). It is important to note, however, that the amount of unique variance explained by these models was very small.

If some models explain unique variance, perhaps combining them would explain more overall variance in face dissimilarity judgments? To address this question, we combined all models into one model via linear weighting and asked whether this combined model explains more variance than each of the models alone. Model weights were assigned within the same procedure that individual models were evaluated, cross-validating over both participants and stimuli. We found that in both datasets, the combined weighted model reached high performance, but did not exceed the performance of the best individual model (Fig. 4*B*).

Models based on BFM or DNN feature spaces outperformed most others, including models based on the face perception literature (angle in the BFM face space and simple configurations of facial features) and two baseline models (based on pixels or 3D face meshes). Poor performance of configural models is at odds with the previously proposed importance of interfeature distances and ratios for face recognition (34–36). It seems that the richer combination of detailed facial landmarks along with their visual appearance as captured by the active appearance model (28) is needed to well predict facial dissimilarity judgments. Such models are also well suited to describing variations across facial expressions (37). The success of the Gabor-based GIST model is broadly consistent with previous work finding that Gabor-based models explain significant variance in face-matching experiments (38, 39) when stimuli are tightly controlled. The GIST model here, however, has high shared variance with more complex models for the image set used, and it is questionable how it would perform under more natural variation of pose and lighting, which we cannot address with the present dataset.

A previous systematic attempt to predict face dissimilarity judgments from image-computable features found that dissimilarity was best predicted by weighted combinations of features that approximated natural high-level dimensions of personal characteristics such as age and weight (40). Here we tested a BFM-based person-attributes model that consisted of the four dimensions of BFM face space that capture the highest variance in height, weight, age, and gender (among the 3D-scanned individuals that BFM space is based on). This model performed significantly and substantially worse than the leading models (*SI Appendix*, Fig. S10).

## Discussion

### BFM Latent Space Predicts Perceived Face Similarity.

We found that Euclidean distance in the principal component latent space of the 3D morphable face-graphics Basel face model is a good approximator of human face dissimilarity judgments. BFM face space supports quantitative predictions of perceived face dissimilarity. This is broadly consistent with a recent report (26) showing that a different shape- and texture-based face-space model is predictive of facial dissimilarity judgments. The model in that study was an active appearance model (27), which like BFM decomposes faces into shape and texture features, but defines shape by landmark locations on 2D face images, not in 3D. The AAM also showed excellent performance in the present study. The Basel 3D morphable model is derived from separate PCAs of 3D face shape and of facial texture and coloration. It is, therefore, a more sophisticated statistical model than the 2D-shape active appearance model and earlier PCA-based face-space models derived from 2D images, which are only moderately predictive of perceived face dissimilarity (41).

Like visual recognition in general, face recognition must be robust to nuisance variation, including variation of view and lighting, but it also has the domain-specific challenge of representing the subtle structural differences between individual faces. It makes sense that our perception of these differences is attuned to the ways in which faces vary in the natural population. Our focus here was on the perceptual representation of the natural statistical variations among faces, rather than on the more general problem of how visual recognition achieves robustness to variation in view, lighting, and other nuisance variables. This is why we sampled a rich set of identities, while keeping view and lighting constant. Using only frontal faces in a single view and lighting condition does make it harder to reject image-computable models, and this has to be taken into account in the interpretation. However, the BFM model, which is central to this study, by definition has perfect invariance to view and lighting and so would have a trivial advantage over the image-computable models if view and lighting were varied. Our approach here gave image-based dissimilarity metrics their best chance to capture the subtle identity-related variations among faces. Future studies varying view and lighting (and perhaps other nuisance variables) along with identity may reveal failures of some of the image-computable models to capture face-identity variation invariantly. It will then be interesting to see whether those failures match or diverge from human face perception.

The success of BFM here is broadly consistent with psychological face-space accounts. However, some previous studies have assigned particular importance to the geometric relationships of faces relative to a meaningful origin of the space (3, 12, 13, 42, 43). Our finding that BFM Euclidean distance uniformly accounts for perceived dissimilarity contrasts with an early report that there are larger perceptual differences between faces that span the average face than between equally BFM-distant faces that fall on one side of the average (44). Extensive behavioral and neurophysiology work has sought to relate the computational mechanisms underlying face perception to geometric relationships in neural or psychological face space. Face-selective neurons may explicitly encode faces in terms of vectors of deviation from an average face. This idea is supported by evidence from monkey electrophysiology (16, 33) and human psychophysical adaptation (3, 17, 45), although alternative interpretations of the latter have been proposed (46, 47). Our comprehensive sampling of face pairs with the full range of possible geometric relationships was tailored to reveal the precise manner in which distances from the origin, and angular distances between faces, affect perceived dissimilarity. Yet both dissimilarity and identity data were best explained by the Euclidean distance. Our results do not contradict previous studies, but suggest that effects of relative geometry may be more subtle than previously thought. We must be mindful also of the fact that the present study sampled faces that vary along diverse dimensions, rather than stimulus sets constructed to densely sample single or few dimensions (e.g., refs. 43, 44). Finally, distances within the BFM appear approximately but not exactly perceptually isotropic.

Distance within the BFM cannot be a perfect predictor of perceived face dissimilarity. First, as mentioned above, BFM latents are perfectly invariant to nuisance variation resulting, for example, from changes in view and lighting, which are known to affect human face perception (48–50). Second, like all morphable models, the BFM cannot account for familiarity with particular individuals, which is known to be a substantial factor in human face perception (42). Third, BFM is based on the head scans of only 200 individuals, and this sample is biased in several ways, for example toward white, relatively young faces. Consistent with these imperfections of BFM distance as a model of human perceptual face space, BFM falls slightly short of reaching the noise ceiling, indicating that it leaves unexplained some of the variance that is reliable across individual observers. It will be interesting to test in the future whether newer 3D morphable face-space models capture more of the remaining variance in human dissimilarity judgments (11) and whether image-computable models can capture human perceptual idiosyncrasies reflecting familiarity with particular individuals as well as human failures of invariance to variation of view and lighting.

### BFM Helps Characterize the Tolerance of Identity Judgments.

For any reference face, BFM face space enables us to approximately define the set of similar faces that people will perceive as the same person. The BFM was previously shown to capture face impressions (51) and personality traits (52), and different kinds of face-space model captured same–different face judgments (23) and the similarity of randomly generated faces to four familiar identities (22). We found that humans often classify pairs of images as depicting the same identity even with relatively large distances in the BFM. Two faces may be perceptibly different from one another, while nevertheless appearing to be “the same person.” The ability of the visual system to generalize identity across some degree of structural difference may be analogous to invariance to position, size, and pose in object recognition (53). Face images generated from a single identity form a complex manifold as they may vary in age, weight, expression, makeup, facial hair, skin tone, and other dimensions (11). Given that we need to robustly recognize identity despite such variation, it may not be surprising that there is a high tolerance for facial feature variation in face recognition. Our stimulus set contained very dissimilar faces, which provided an anchor for people’s definition of “different” and may influence moderately dissimilar faces to look quite alike, in comparison. Participants seemed to interpret person identity quite generously, possibly imagining whether this face could be the same person if the person aged, got tanned, or lost weight. “The same person” may be a not precisely defined concept; however, people seem to agree what that concept means as they were consistent in judging the same/different identity boundary. Interestingly, the “different identity” threshold was not far from the saturation point of face dissimilarity psychometric function (Fig. 3*A*). This result could be related to people dismissing all “different individuals” as completely different and focusing their sensitivity within the range of faces that could depict the same identity. The design of our current experiment required participants to locate a threshold of dissimilarity, above which they considered faces to be different identities. This means that identity and face dissimilarity judgments are constrained to be consistent. Future experiments could be designed to enable subjects to judge one pair as dissimilar, yet same identity, and another as more similar, yet different in identity.

### Multiple Models Predict Human Judgments Similarly Well.

Our data show clearly that some models of face dissimilarity are worse than others. Simply taking the angle between faces in the BFM is a poor predictor, as is a set of higher-order ratios between facial features. Perhaps surprisingly, the model consisting only of the four dimensions that capture the highest variance in height, weight, age, and gender performed poorly. It is possible that this model did not perform optimally because the face pair images in the face dissimilarity judgments did not have enough variability across these dimensions. Age and gender were shown to explain variance in face magnetoencephalography (MEG) representations (54) and we show that they do explain variance in the face dissimilarity judgments task, however, to a lesser extent than other models. It seems that people use other or more than socially relevant dimensions when judging face dissimilarity.

Among highly performing models, we found that several explain face dissimilarity judgments similarly well. One of the models that explains a surprisingly large amount of variance is GIST. It has been previously shown that Gabor-based models explain face representations well (24, 38). The models compared contain quite different feature spaces. For example, object-trained and face-trained VGG models learn distinctly different feature detectors (7), yet explain a similar amount of variance in human face dissimilarity judgments. Object-trained and face-trained VGG models have previously been found to explain a similar amount of variance in human inferior temporal cortex (55) and in face-selective visual cortex (56), and object-trained VGG captured variance in early MEG responses (57). The face space within a face-trained DNN organizes faces differently than they are arranged in the BFM’s principal components, for example, clustering low-quality images at the “origin” of the space, eliciting lower activity from all learned features (42).

One of the reasons for similarly high performance among disparate models is that, for our stimulus set, several models made highly correlated predictions (*SI Appendix*, Fig. S12), making it difficult to discriminate between them based on the current data. Model discrimination was also difficult in a previous study of face representations in the human fusiform face area, where a model based on Gabor filters performed similarly to one based on sigmoidal ramp tuning in face space (24). It is important to bear in mind that stimulus sampling strategies can affect the relative performances of different models. If stimuli vary only along a subset of the perceptual dimensions, then the experiment cannot reveal to what extent each model explains the other dimensions. Here we specifically sampled face pairs in BFM, which suggests that our experiment should powerfully reveal the strengths and weaknesses of BFM, while possibly missing other models’ strengths and/or weaknesses along dimensions not varied in our stimulus set. Our stimulus set, thus, is biased in that it samples BFM latent space. However, it is unclear how an unbiased stimulus set should be defined and whether our stimulus set here helps or hurts BFM’s performance relative to other models. In fact, we observed that several models including AAM and DNNs performed very well in explaining face similarity data despite being derived by training on very different face information and sampling strategies. Future studies could use stimulus optimization methods to identify sets of stimuli for which candidate models make divergent predictions (58–60).

The models we considered here are distributed over a vast space, and it is not possible in a single study to probe all models’ representational spaces adequately. The BFM and image-computable models have different advantages and disadvantages for predicting human dissimilarity judgments. The BFM model has privileged access to distances in a shape and texture space normalized to represent the distribution of natural faces. However, the image-computable models have privileged access to image features of the kind known to be represented throughout the ventral visual stream. Similarly, the 3D mesh model has privileged access to the shape of the face surfaces. Comparing many models provides constraints for computational theory, but it will take many studies to drive the field toward a definitive computational model of face perception. We conclude that in addition to being a useful tool for generating faces in face perception research, statistical generative models of face shape and texture align surprisingly well with human face perception.

## Materials and Methods

### Stimuli.

Each face generated by the BFM corresponds to a unique point in the model’s 398-dimensional space (199 shape dimensions and 199 texture dimensions), centered on the average face. The relative locations of any pair of faces can therefore be summarized by three values: the length of the vector from the origin to the first face *r*_1_, the length of the vector from the origin to the second face *r*_2_, and the angle between the two face vectors *θ* (Fig. 1*B*). To create a set of face pairs spanning a wide range of relative geometries in face space, we systematically sampled all pairs of eight possible radius values combined with eight possible angular values. Possible angular values were eight uniform steps between 0 and 180^∘^, and possible vector lengths were eight uniform steps between 0 and 40 units in the BFM. The measure of 40 units corresponds to 40 SDs. For reference, the distance from a randomly sampled point to the origin of the space, when drawing randomly from a 398-dimensional space where each dimension is independently normally distributed with unit variance, is 19.95 SDs (i.e., the square root of 398) (14). Our goal was to extensively sample face space, including faces that lie near the expected distance from the origin, as well as faces closer to or farther from the origin than would be expected by chance. This sampling procedure led some faces far away from the origin to look slightly caricatured. However, caricatured faces are in the minority, and we find they are fairly evenly distributed among pairs, appearing in pairs rated as looking both very dissimilar and very similar (both full stimulus sets are provided in *SI Appendix*, Figs. S2 and S3). For all eight angles and eight eccentricities, there were 232 unique face relationships when considering 1) that exchanging the two radii yields the same relationship for a given angle and 2) that the angle is irrelevant when one of the radii is 0. When both radii are >0, there are (7[nonzero radii] × 7[nonzero radii] + 7)/2 = 28 pairs of nonzero radius combinations (including identical radii), and so there are 28 × 8[angles] = 224 relationships between faces. When one of the radii is 0, then the angle is irrelevant, and there are an additional eight radius combinations (the other radius can take each of the eight values). For each relative geometry, we then sampled two random points in the full 398-dimensional BFM space that satisfied the given geometric constraints. We generated two separate sets of face pairs with the same relative geometries but different face exemplars, by sampling two independent sets of points satisfying the same geometric constraints. The two sets (stimulus set A and stimulus set B) were used as stimuli in separate experimental sessions (*Psychophysical Face-Pair Arrangement Task*).

### Participants.

Human behavioral testing took place over three sessions. Twenty-six participants (13 females) took part in sessions 1 and 2, and a subset of 15 (6 females) took part in session 3. All testing was approved by the Medical Research Council (MRC) Cognition and Brain Sciences Ethics Committee and was conducted in accordance with the Declaration of Helsinki. Volunteers gave informed consent and were reimbursed for their time. All participants had a normal or corrected-to-normal vision.

### Psychophysical Face-Pair Arrangement Task.

The procedure in all sessions was identical, the only difference being that the same set of face-pair stimuli was used in sessions 1 and 2, while session 3 used a second sampled set with identical geometric properties. Comparing the consistency between sessions 1, 2, and 3 allowed us to gauge how strongly human judgments were determined by geometric relationships in face space, irrespective of the individual face exemplars.

During an experimental session, participants were seated at a comfortable distance in front of a large touchscreen computer display (43-in. Panasonic TH-43LFE8-IR, resolution 1,920 × 1,080 pixels, touchscreen size 96.9 × 56 cm) placed horizontally on a desk (participants looked at faces from above). On each trial, the participant saw a large white “arena,” with a randomly arranged pile of eight face pairs in a gray region to the right-hand side (Fig. 1*C*). The two faces within each pair were joined together by a thin line placed behind the faces, and each pair could be dragged around the touchscreen display by touching. Each face image was rendered in color with a transparent background and a height of 144 pixels ( ∼7.1 cm on screen).

The bottom edge of the white arena was labeled “identical” and the top edge was labeled “maximum difference.” Two example face pairs were placed to the left and to the right of the identical and maximum difference labels to give participants reference points on what identical and maximally different faces look like. The maximally different example faces had the largest geometric distance possible within the experimentally sampled geometric relationships (i.e., the Euclidean distance in the BFM = 80) in contrast to identical faces (i.e., the Euclidean distance in the BFM = 0). The same example pairs were used for all trials and participants.

Participants were instructed to arrange the eight face pairs on each trial vertically, according to the dissimilarity of the two faces within the pair. For example, two identical faces should be placed at the very bottom of the screen. Two faces that look as different as faces can look from one another should be placed at the very top of the screen. Participants were instructed that only the vertical positioning of faces would be taken into account (horizontal space was provided so that face pairs could be more easily viewed and so that face pairs perceived as being equally similar could be placed at the same vertical location). On each trial, once the participant had finished vertically arranging face pairs by dissimilarity, they were asked to drag an “identity line” (Fig. 1*C*) on the screen to indicate the point below which they considered image pairs to depict the same person. Once eight face pairs and the identity line were placed, participants pressed the “done” button to move to the next trial. Each session consisted of 29 trials.

### Representational Similarity Analysis.

We used representational similarity analysis (RSA) to evaluate how well each of a set of candidate models predicted human facial (dis)similarity judgments (61). For every model, a model-predicted dissimilarity was obtained by computing the distance between the two faces in each stimulus pair, within the model’s feature space, using the model’s distance metric (*Candidate Models of Face Dissimilarity*). Model performance was defined as the Pearson correlation between human dissimilarity judgments and the dissimilarities predicted by the model. We evaluated the ability to predict human data both for each individual model and for a linearly weighted combination of all models (62, 63). See *SI Appendix* for details.

### Area under the Receiver Operating Characteristic Curve Calculation.

We calculated the receiver operating characteristic (ROC) curve such that for each Euclidean distance, *θ* or absolute difference between radii we first run a logistic regression (classifier) to predict identity (based on position relative to identity line). Second, we computed the ROC curve on the classifier output. Specifically, for a range of discriminatory thresholds (0 to 1), we computed the true positive rate and false positive rate. The ROC curve is the plot of true positive rate vs. false positive rate. The area under the curve (AUC) represents how well the classifier performs at various threshold settings. The threshold is the value at which the classifier will assign a label to a given input, by comparing the probability of that input vs. the threshold.

### Unique Variance Analysis.

We used a general linear model (GLM) to evaluate unique variance explained by the models (64). For each model, unique variance was computed by subtracting the total variance explained by the reduced GLM (excluding the model of interest) from the total variance explained by the full GLM. For model m, we fit GLM on X = “all models but m” and Y = data, and then we subtract the resulting *R*^2^ from the total *R*^2^ (fit GLM on X = “all models” and Y = data). We performed this procedure for each participant and used nonnegative least squares to find optimal weights. A constant term was included in the GLM. We performed a one-sided Wilcoxon signed-rank test to evaluate the significance of unique variance contributed by each model across participants.

### Candidate Models of Face Dissimilarity.

We considered a total of 16 models of face dissimilarity in the main analyses (Fig. 4) and an additional 3 models in *SI Appendix*, Fig. S10. Each model consists of a set of features derived from a face image, BFM coordinates, or 3D mesh, combined with a distance metric (Table 1).

**Table 1.** Candidate models of face dissimilarity

Model name	Description	Source	Distance metric	Computed from
1) BFM	3D morphable face model combining PCA subspaces of structural and textural components from 200 3D face scans.	(10)	Euclidean	BFM PCA coordinates
2) BFM angle	PCA subspace of only the structural components from 200 3D face scans.	(10)	Cosine	BFM PCA coordinates
3) BFM shape (*SI Appendix*, Fig. S10)	PCA subspace of only the structural components from 200 3D face scans.	(10)	Euclidean or cosine	BFM PCA coordinates
4) BFM texture (*SI Appendix*, Fig. S10)	PCA subspace of only the textural components from 200 3D face scans.	(10)	Euclidean or cosine	BFM PCA coordinates
5) BFM person attributes (*SI Appendix*, Fig. S10)	Loading of PCA coordinates onto height, weight, age, and gender vectors.	(10)	Euclidean or cosine	BFM PCA coordinates
6) VGG-Face best	Highest-performing layer of 16-layer deep neural network trained on face identification.	(30)	Euclidean	RGB image
7) VGG-Object best	Highest-performing layer of 16-layer deep neural network trained on object recognition.	(18)	Euclidean	RGB image
8) VGG–BFM-identity best	Highest-performing layer of 16-layer deep neural network trained on BFM synthetic identity recognition.	Custom trained	Euclidean	RGB image
9) VGG–BFM-latents best	Highest-performing layer of 16-layer deep neural network trained on BFM images to infer BFM latent parameters.	Custom trained	Euclidean	RGB image
10) AlexNet best	Highest-performing layer of 8-layer deep neural network trained on object recognition.	(31)	Euclidean	RGB image
11) Active appearance model	Shape and appearance description in active appearance model.	(28)	Euclidean	RGB image
12) Eigenfaces	Face space comprising all eigenvectors results from a PCA on 5,000 face photographs.	(66, 67)	Euclidean	RGB image
13) HMAX best	Highest-performing layer in a 4-layer cortically inspired neural network.	(68)	Euclidean	RGB image
14) GIST	Gabor-based summary of contrast energy at different orientations and scales.	(69)	Euclidean	RGB image
15) Pixel	Raw image data.	n/a	Euclidean	RGB image
16) Mesh	Raw 3D mesh data.	n/a	Euclidean	3D mesh
17) Configural 0th	Locations of key facial features (0th-order configural information).	(35)	Euclidean	3D mesh
18) Configural 1st	Distances between key facial features (first-order configural information).	(35)	Euclidean	3D mesh
19) Configural 2nd	Ratios of distances between key facial features (second-order configural information).	(35)	Euclidean	3D mesh

n/a, not applicable.

#### Basel face model.

We considered two variant models based on the principal component space provided by the BFM: 1) “BFM Euclidean,” which took the Euclidean distances between faces in the full 398-dimensional BFM space, and 2) “BFM angle,” which took the cosine distance between face vectors in the full 398-dimensional space. See *SI Appendix* for details.

#### Active appearance model.

Active appearance models (27) are 2D morphable models, generally trained on hand-annotated face images to identify a number of key facial landmarks from 2D photographs and describe visual structure around these landmarks. We applied a pretrained AAM provided in the Menpofit Python package (https://www.menpo.org/menpofit/) (28) as menpofit.aam.pretrained. The model is a patch-based AAM that has been trained on 3,283 face photographs hand labeled with the locations of 68 landmark features (https://ibug.doc.ic.ac.uk/resources/facial-point-annotations/). The model describes the shape of a face via 20 shape parameters that summarize the locations of landmark features and the texture of a face via 150 appearance parameters that summarize pixel values in the form of dense scale-invariant feature transform features. We used a Lucas–Kanade AAM fitter provided in the Menpofit package to fit the parameters of the pretrained model to each of our stimulus faces independently. We provided an initial guess via a 72 × 72-pixel bounding box at the center of each face image and then ran the fitting procedure for 100 iterations. An example fitting result is shown in *SI Appendix*, Fig. S13*A*. After fitting the AAM to each stimulus face we appended the final shape and appearance parameters to create a 170-dimensional vector describing the face, analogously to how shape and texture PCs were appended to create the full Basel face model descriptor. We then took the Euclidean distance between vectors as the predicted dissimilarity between faces in each stimulus pair.

#### Models based on 3D face structure.

Face perception is widely thought to depend on spatial relationships among facial features (5, 34, 35, 65). We calculated the Euclidean distance between the 3D meshes that were used to render each face (“mesh” model). We also used the geometric information within each face’s mesh description to calculate a first-, a second-, and a third-order configural model of facial feature arrangements, following suggestions by ref. 35 and others (e.g., ref. 34) that face perception depends more strongly on distances or ratios of distances between facial features than raw feature locations. See *SI Appendix* for details.

#### Deep neural networks.

We used a pretrained state-of-the-art 16-layer convolutional neural network (VGG-16), trained on millions of images to recognize either object classes (30) or facial identities (18). Further details can be found in refs. 18 and 30. The dissimilarity predicted by DNN models was defined as the Euclidean distance between activation patterns elicited by each image in a face pair in a single layer. To input to DNN models, faces were rendered at the VGG network input size of 224 × 224 pixels, on a white background, and preprocessed to subtract the average pixel value of the network’s training image set. See *SI Appendix* for details about the VGG–BFM-identity classification network and the VGG–BFM-latents regression network.

#### Low-level image-computable models.

As control models, we also considered the dissimilarity of two faces in terms of several low-level image descriptors: 1) Euclidean distance in raw RGB pixel space; 2) Euclidean distance within a “GIST” descriptor, image structure at four spatial scales and eight orientations (https://people.csail.mit.edu/torralba/code/spatialenvelope/); 3) Euclidean distance within the best-performing layer (C2) of HMAX, a simple four-layer neural network (cbcl.mit.edu/jmutch/hmin/); and 4) Euclidean distance within an “Eigenface” space (66) consisting of the 4,999 dimensions obtained by running a PCA on a “training dataset” comprising the first 5,000 faces in the CelebA cropped and aligned dataset of celebrity faces (mmlab.ie.cuhk.edu.hk/projects/CelebA.html) (67). To create the PCA space, photographs were resized to a height of 144 pixels and then cropped to the center 144 × 144 pixels. To derive predicted dissimilarities for experimental stimuli, we first subtracted the mean image from the PCA training dataset from each stimulus face and then projected the stimulus face into the 4,999-dimensional PCA space to obtain a description of each face in terms of a 4,999-dimensional weight vector. We took the Euclidean distance between projected PCA weight vectors as the predicted dissimilarity between face images. Experimental stimuli could be well reconstructed from weighted combinations of Eigenfaces, as shown in *SI Appendix*, Fig. S13*A*. For comparability with the images seen by participants, all low-level image-computable models operated on faces rendered on a white background at 144 × 144-pixel resolution.

## Supplementary Material

Supplementary File

## Data Availability

Anonymized human behavioral judgment data have been deposited in The Open Science Framework (https://osf.io/7bh6s/). The code to analyze the raw behavioral data and replicate the figures in the paper can be found at GitHub, https://github.com/kamilajozwik/face_similarity_paper_2022.
