# Supplementary material for: Face dissimilarity judgments are predicted by representational distance in morphable and image-computable models
Source: Proc Natl Acad Sci U S A. 2022 Jun 29;119(27):e2115047119. doi: 10.1073/pnas.2115047119 (PMC9271164; doi:10.1073/pnas.2115047119)
Supplement: Supplementary File [file pnas.2115047119.sapp.pdf]

Supplementary Materials

Supplementary Methods

Representational similarity analysis. To provide an estimate of the upper bound of explainable variance in the dataset, we calculated how well human data could be predicted by data from other participants, providing a "noise ceiling". Noise ceilings, raw model performance, sigmoidally-transformed model performance, and reweighted combined model performance were all calculated within a single procedure, cross-validating over both participants and stimuli. On each of 30 cross-validation folds, 5 participants and 46 face pairs were randomly assigned as test data, and the remaining stimuli and participants were used as training data. On each fold, a sigmoidally-transformed version of each model was created, by fitting a logistic function to best predict dissimilarities for training stimuli, averaged over training participants, from raw model distances. Also on each fold, a reweighted combined model was created using non-negative least-squares to assign one positive weight to each of the individual models, to best predict the dissimilarity ratings for training stimuli, averaged over training participants. We then calculated, for each raw model, each sigmoidally transformed model, and for the combined reweighted model, the Pearson correlation with the model's predictions for test stimuli for each individual test participant's ratings. The average correlation over test participants constituted that model's performance on this cross-validation fold. The upper bound of the noise ceiling was calculated within the same fold by correlating each test participant's test-stimulus data with the average test-stimulus data of all test participants (including their own). The lower bound was calculated by correlating each test participant's test-stimulus data with the average for all training participant's test-stimulus data. Means and confidence intervals were obtained by bootstrapping the entire cross-validation procedure 2,000 times over both participants and stimuli.

We first determined whether each model was significantly different from the lower bound of the noise ceiling, by assessing whether the 95% confidence interval of the bootstrap distribution of differences between model and noise ceiling contained zero, Bonferroni corrected for the number of models. Models that are not significantly different from the lower bound of the noise ceiling can be considered as explaining all explainable variance, given the noise and individual differences in the data. We subsequently tested for differences between the performance of different models. We defined a significant pairwise model comparison likewise as one in which the 95% confidence interval of the bootstrapped difference distribution did not contain zero, Bonferroni corrected for the number of pairwise comparisons.

Basel Face Model. For face pairs where cosine distance was undefined, because one face lay at the origin of BFM space, the angle between the two faces was defined as zero for the purposes of model evaluation. To more fully explore the relationship between apparent dissimilarity and placements of faces in the full BFM space, we also considered linear and sigmoidal functions as candidates for predicting the relationship between the Euclidean distance in the BFM and face dissimilarity judgements. We estimated each model's predictive performance as the Pearson correlation between the fitted model's predicted dissimilarities and the dissimilarities reported by each participant. We tested for significant differences between linear and sigmoidal function fits using a two-sided Wilcoxon signed-rank test. For each participant, we fitted the model to half of the data (session 1) and measured the predictive accuracy of the model in the second half of the data (session 2). The predictive accuracies were averaged across participants.

Finally, the BFM provides the axes onto which the height, weight, age, and gender of the 3D scanned participants most strongly loads. By projecting new face points onto these axes, we can approximately measure the height, weight, age and gender of each generated face. The "Person attributes" model consisted of the Euclidean distance between faces, after projecting faces onto these four dimensions.

Models based on 3D face structure. We selected 30 vertices on each face corresponding to key locations such as the centre and edges of each eye, the edges of the mouth, nose, jaw, chin, and hairline, using data provided in the BFM. The positions of these 30 vertices on each 3D face mesh formed the features for the "0th order" configural model. We then calculated 19 distances between horizontal and vertically aligned features (e.g. width of nose, length of nose, separation of eyes), which formed the "1st order" configural model. Finally, we calculated 19 ratios among these distances (e.g. the ratio of eye separation to eye height; the ratio of nose width to nose length), which formed the "2nd order" configural model. For all configural models, the predicted dissimilarity between two faces was the Euclidean distance between their respective feature vectors.

Deep neural networks. We formed the VGG-BFM-identity classification network by training the VGG-16 architecture (1, TorchVision's implementation) to classify Basel Face Model (2) face images of 8,631 synthetic identities (Supplementary Figure 14). All of the images pertaining to one identity shared shape and texture latents (both randomly sampled from the Basel Face Model, once per identity), but had different expression latents, poses, lighting direction, and lighting intensity. We generated 363 images of each identity to roughly match the total number of training images in the VGGFace2 dataset (3). The rendered images were randomly cropped during training or centre-cropped during validation, in both cases yielding an input image of 224 x 224 pixels. To further increase the images' variability, we augmented the training examples using Albumentations (4). We only included naturalistic transformations such as grayscale transformation, brightness and contrast manipulations, noise addition, drop-out, grid distortion, and blurring. See Supplementary Figure 16a for training-set image examples. The input images were normalized by the channel-specific mean and standard deviation, computed from a subset of training images. The model was trained for 30 epochs on

minimizing the cross-entropy loss, using four GPUs. We used stochastic gradient descent with a weight decay of 0.0001, momentum of 0.9, and a minibatch size of 512. The learning rate was initialised to 0.01 and reduced by a factor of 10 every 10 epochs. The model reached a 0.0 validation loss.

We formed the VGG-BFM-latents regression network by mapping the penultimate layer of a VGG-16 architecture to a 508-dimensional vector as the last fully connected layer and training the network to recover the underlying latents of synthetic face images sampled from the Basel Face Model (2, Supplementary Figure 15). 199 of the 508 output units were assigned as predicted shape coefficients, 199 as predicted texture coefficients, 100 as predicted expression coefficients, 4 as predicted face pose (parameterized as a quaternion), 3 as lighting color, and 3 as lighting direction. The network was trained on minimizing the sum of six normalized mean squared error (NMSE) terms (i.e., shape, texture, expression, face pose, ambient lighting, and lighting direction). The synthetic dataset included 3,300,0000 unique faces generated similarly to the dataset used to train the VGG-BFM-identity model, except that each face was independently sampled from the BFM (i.e., without using synthetic identities) and we did not use dataset augmentations other than random cropping. The model was trained for 120 epochs using four GPUs. We used ADAM (5) with $\beta_0 = 0.9$, $\beta_1 = 0.999$, $\epsilon = 10^{-8}$, no weight decay, and a minibatch size of 512. The learning rate was initialised to 0.0001 and was reduced by a factor of 10 every 40 epochs. See Supplementary Figure 16b,c for quantitative and qualitative evaluation of the trained model's performance.

We used PyTorch (6) for implementing the VGG-BFM-identity and VGG-BFM-latents models and PyTorch Lightning (www.pytorchlightning.ai) for model training.

References

1. K Simonyan, A Zisserman, Very deep convolutional networks for large-scale image recognition. arXiv (2014).
2. T Gerig, et al., Morphable Face Models - An Open Framework. IEEE Int. Conf. on Autom. Face Gesture Recognit. pp. 75–82 (2018).
3. OM Parkhi, A Vedaldi, A Zisserman, Deep face recognition. arXiv (2015).
4. A Buslaev, A Parinov, E Khvedchenya, VI Iglovikov, AA Kalinin, Albumentations: fast and flexible image augmentations. Information 11, 125 (2020).
5. DP Kingma, J Ba, Adam: A method for stochastic optimization. in 3rd International Conference on Learning Representations (2015).
6. A Paszke, et al., Automatic differentiation in pytorch. in NIPS-W (2017).

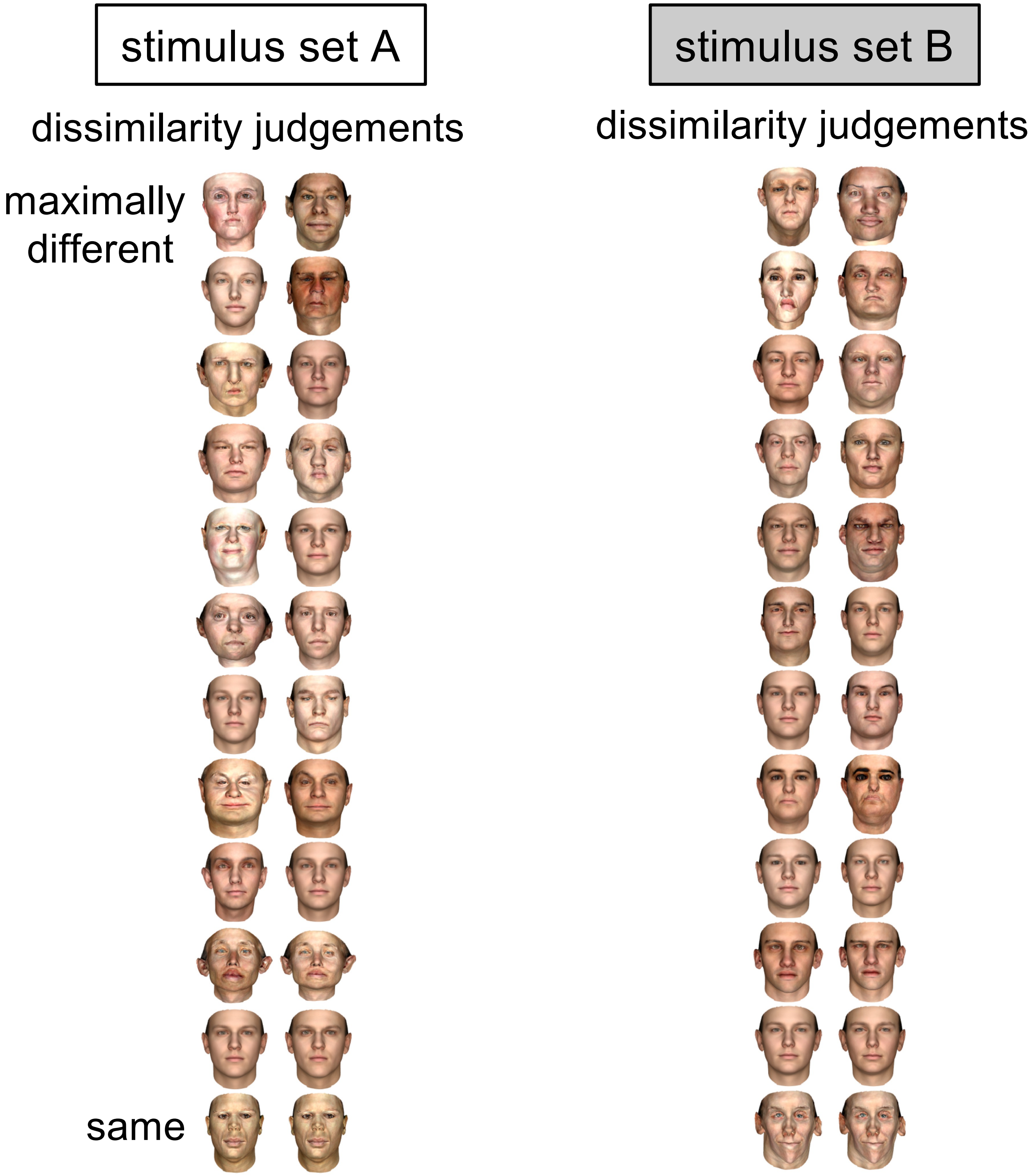

Supplementary Fig. 1. Face dissimilarity rankings by humans.
Columns display face pairs according to their average rated dissimilarity by humans from most dissimilar (top) to most similar (bottom), visualising every 20th face pair in each ranked set within stimulus sets A (left) and B (right).

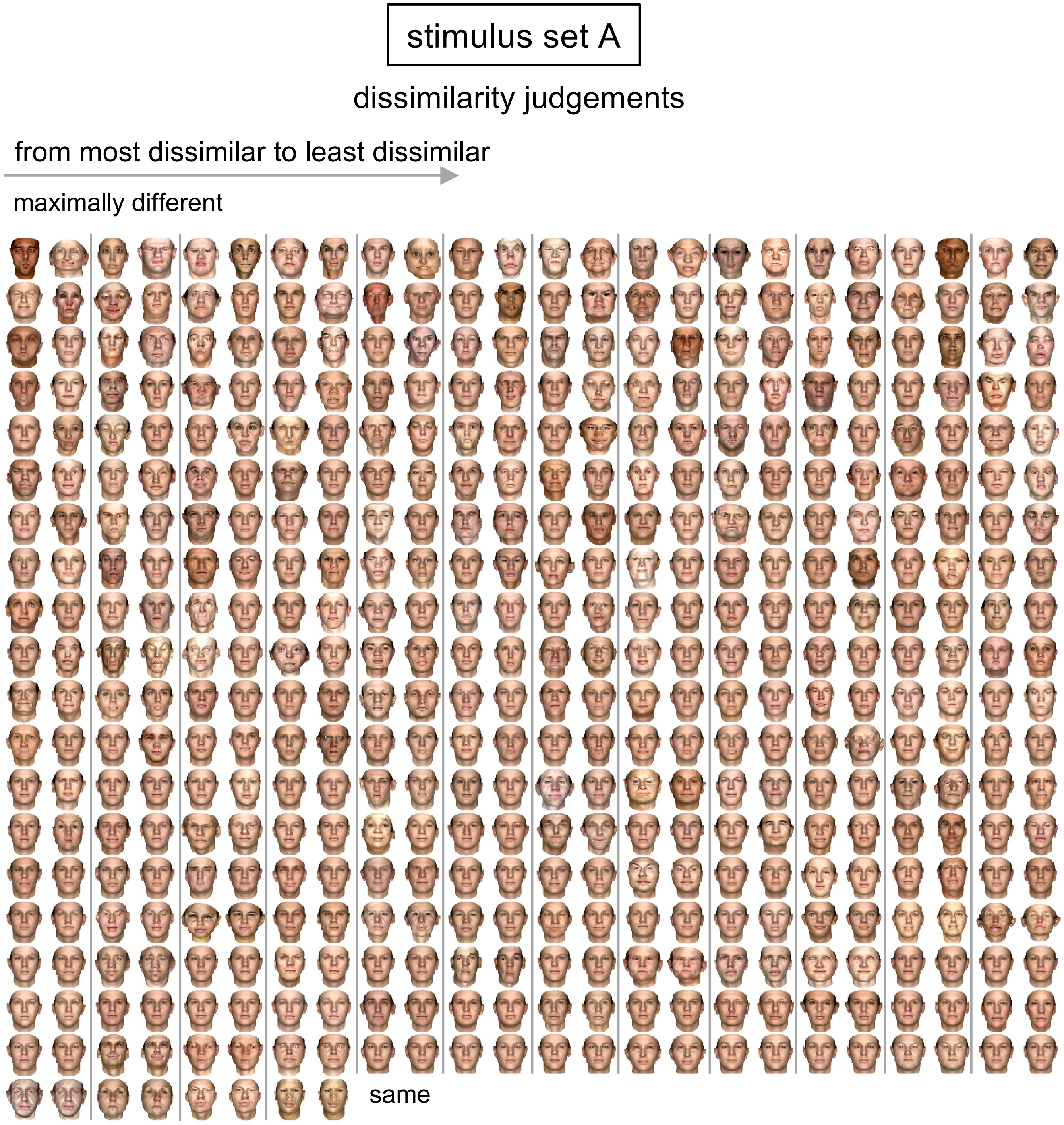

Supplementary Fig. 2. Face dissimilarity rankings by humans for all face pairs within stimulus set A.
Columns display face pairs ordered according to their average rated dissimilarity by humans from most dissimilar (top left) to most similar (bottom right).

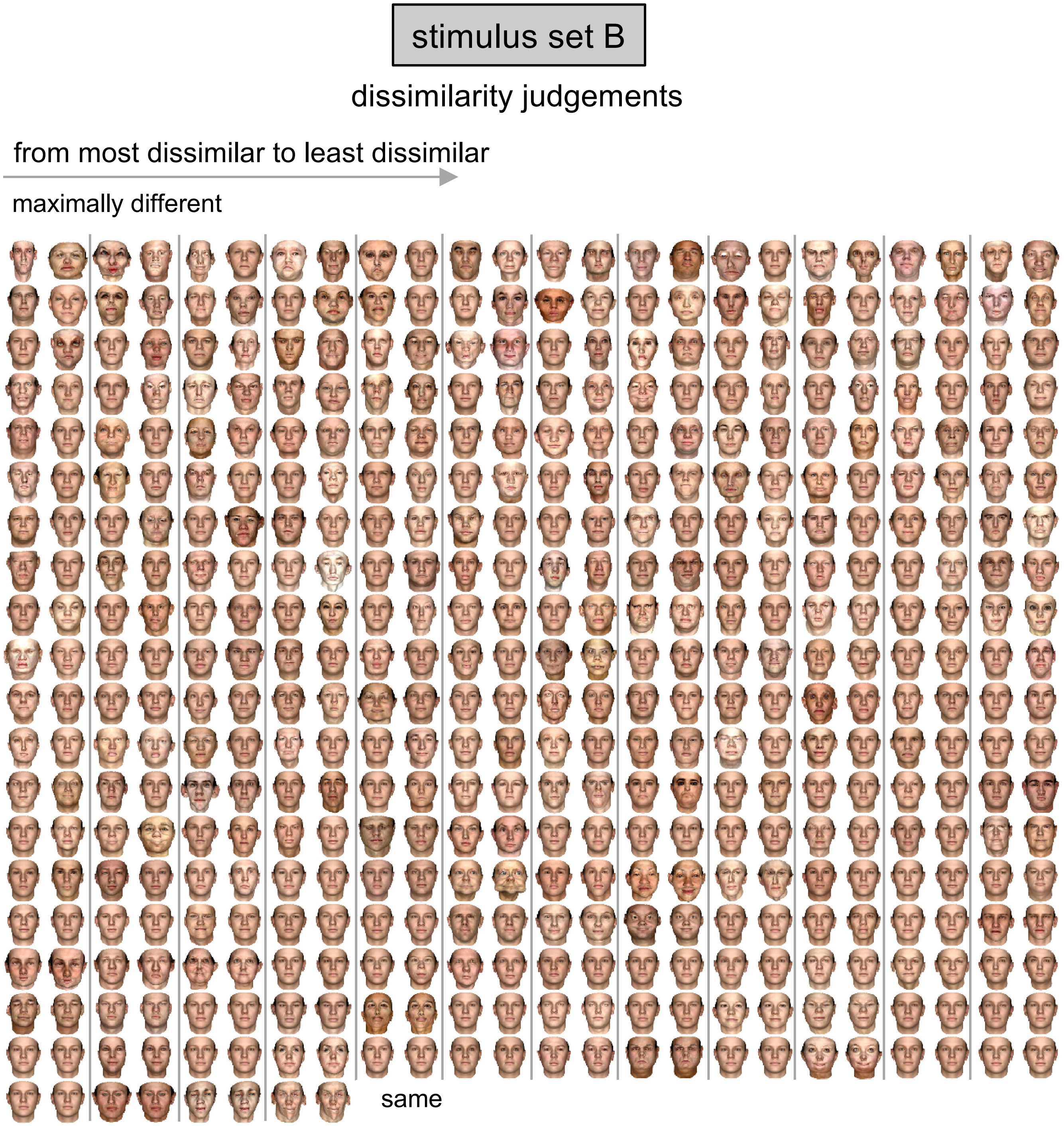

Supplementary Fig. 3. Face dissimilarity rankings by humans for all face pairs within stimulus set B.
Columns display face pairs ordered according to their average rated dissimilarity by humans from most dissimilar (top left) to most similar (bottom right).

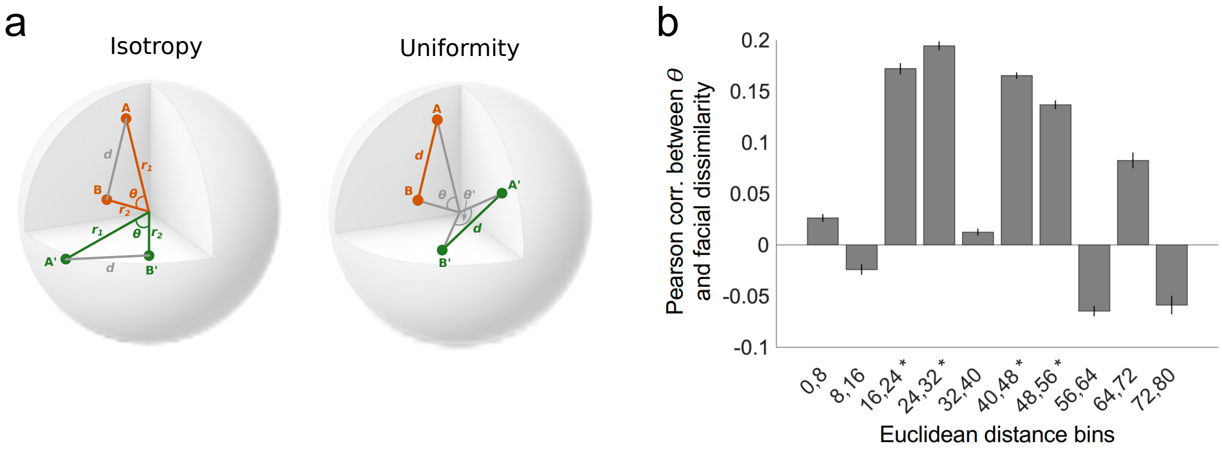

Supplementary Fig. 4. Uniformity test.

a) Schematic of the distinction between perceptual isotropy and perceptual uniformity. If a space is perceptually isotropic (left), the pairs of faces (A,B) and (A',B') should appear equally dissimilar, because they correspond to vectors that have been rotated around the origin while preserving their geometric relationship to one another (the vectors span the same angle θ and have the same norms r_1 and r_2). If a space is perceptually uniform (right), the pairs of faces A-B and A'-B' should appear equally dissimilar, because they correspond to vectors that have been linearly translated in the space, preserving their Euclidean distance to one another (while disrupting their geometric relationship).

b) Analysis evaluating evidence of perceptual uniformity in the stimulus set A experiment. Face pairs were binned into groups with similar Euclidean distances. We then evaluated whether the angle between the faces explains variance in perceived dissimilarity within each bin. If the space is non-uniform, we might expect faces with larger angular differences to appear more different, even if they have identical Euclidean distance. We find only weak evidence for any non-uniformity in the face space. Bins with significant correlation are indicated by an asterisk (one-sided Wilcoxon signed-rank test, $P < 0.05$ corrected). Error bars show the standard error of the mean based on single-participant correlations.

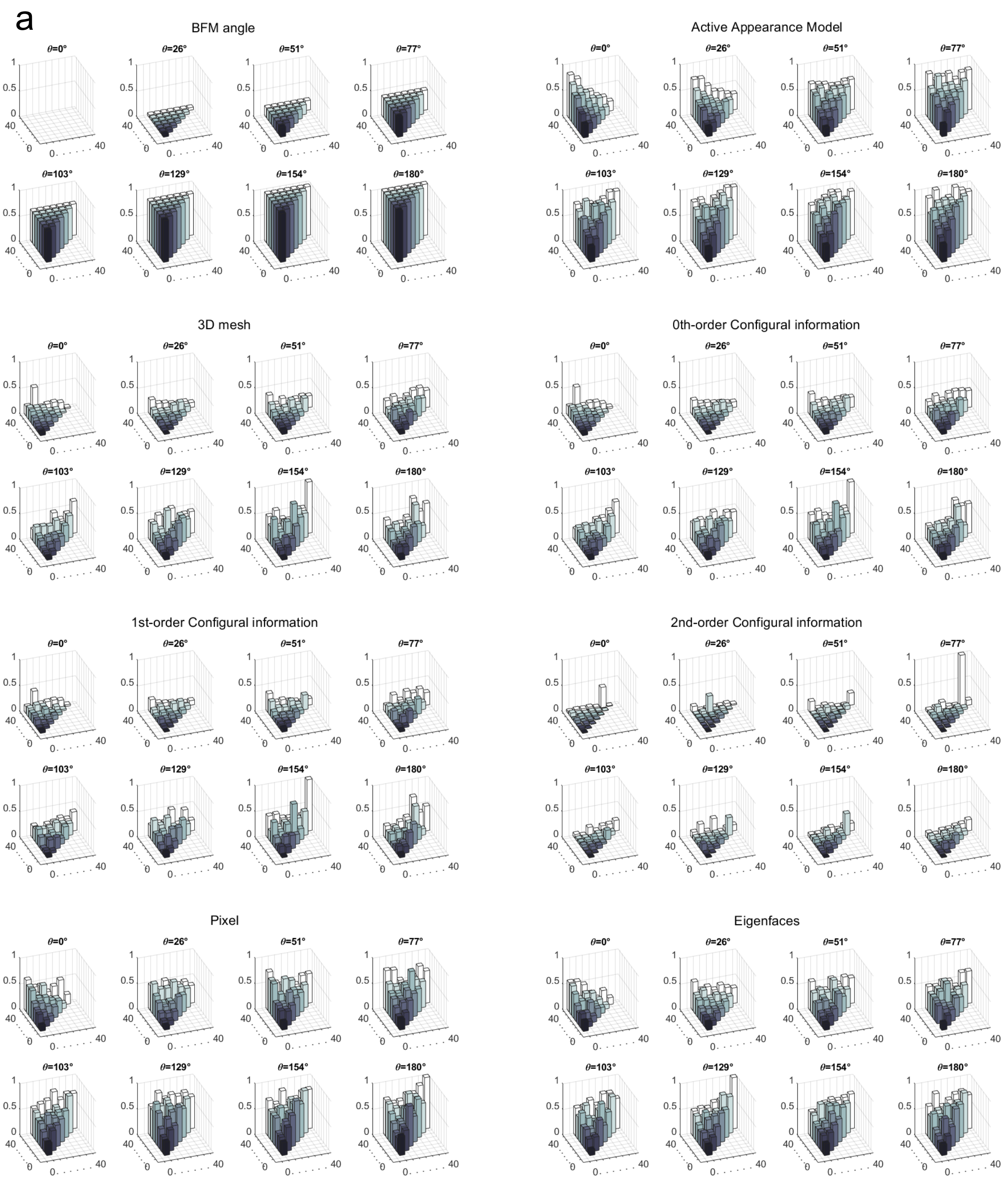
a
BFM angle
Active Appearance Model
3D mesh
0th-order Configural information
1st-order Configural information
2nd-order Configural information
Pixel
Eigenfaces
θ=0°
θ=26°
θ=51°
θ=77°
θ=103°
θ=129°
θ=154°
θ=180°

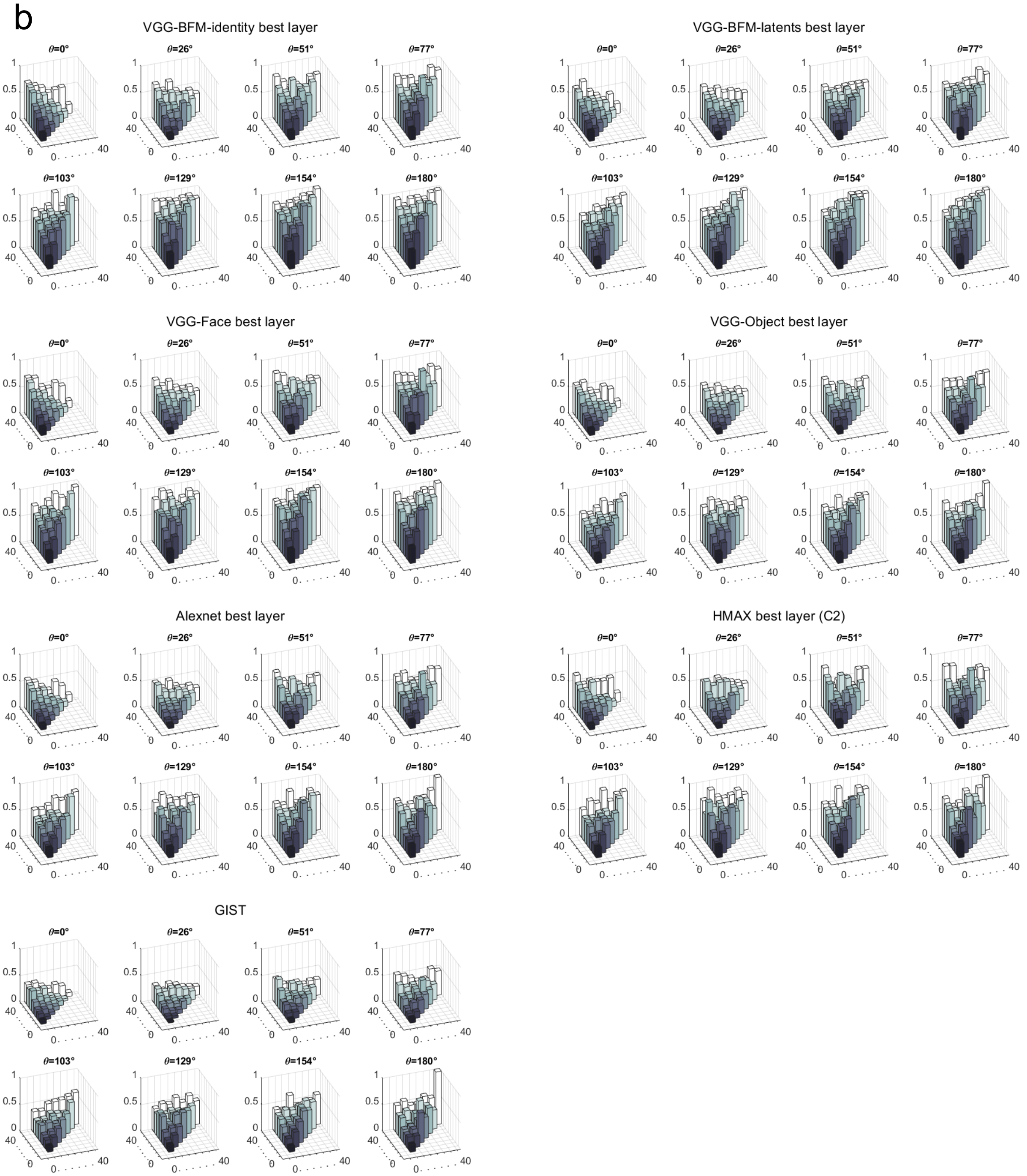

Supplementary Fig. 5. Dissimilarity predictions for each face pair in the stimulus set A as a function of angular and radial geometry in BFM, according to each model.
a) Models based on BFM information, facial geometry, landmarks or configurations, and simple 2D image properties.
b) Models based on deep neural network representations or shallower computer vision features. Conventions are as in Figure 2a of the main manuscript. Each plot shows a "slice" through the BFM face space, comprising stimuli separated by the same BFM angle. The x and y axes indicate the length of the longer and shorter radius in the face pair, and the height of the bar indicates predicted dissimilarity for the face pair according to the model, normalised to the range 0-1.

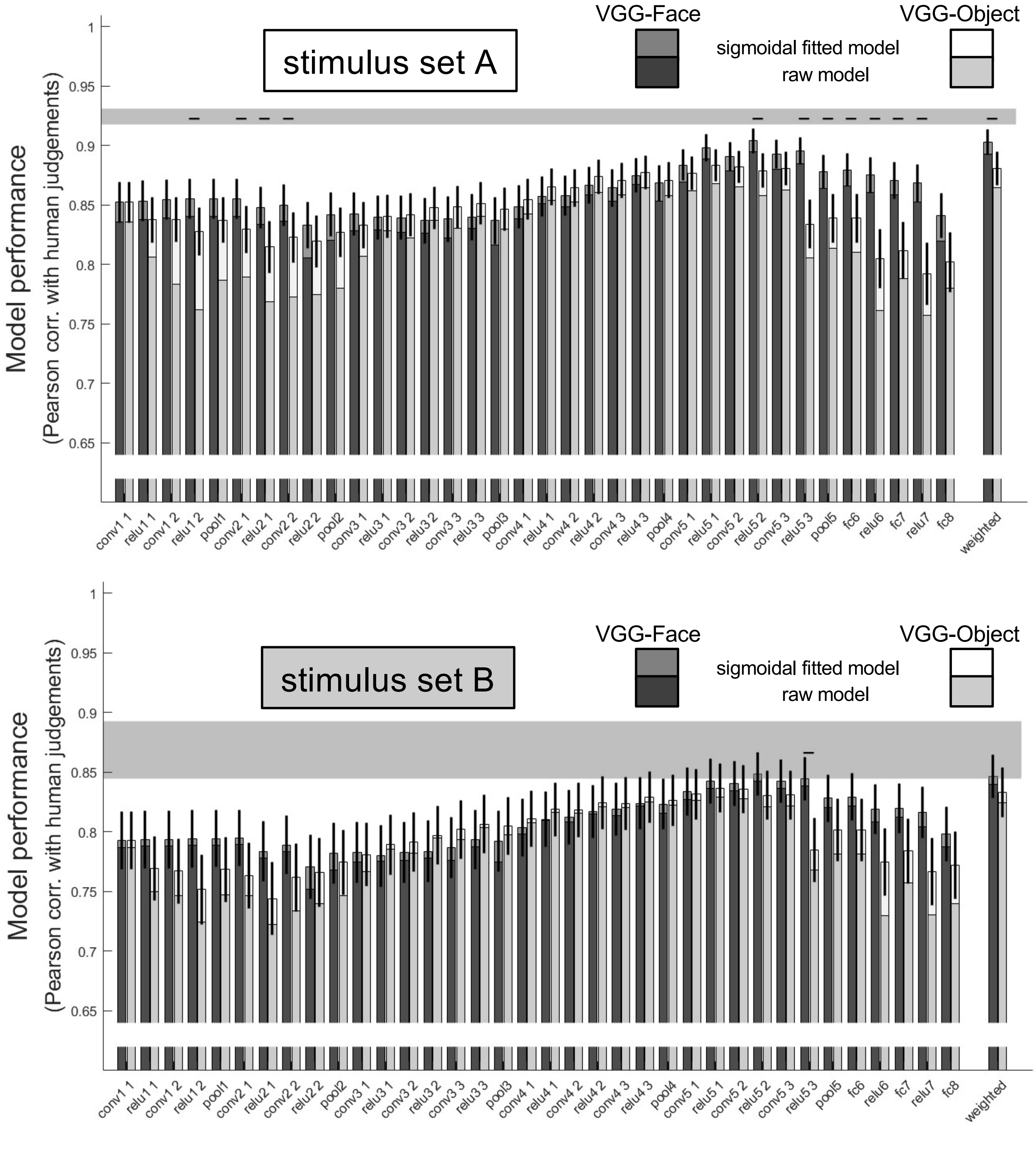

Supplementary Fig. 6. Ability of each layer within VGG-Face and VGG-Object neural networks to predict human face dissimilarity judgements.
Correlations between human dissimilarity judgements in stimulus sets A (top) and B (bottom) and Euclidean distance within each layer of a deep neural network trained either to recognise faces (VGG-Face) or objects (VGG-Object). All key processing steps within each network are shown, including application of a non-linearity ('relu'), convolutional layers ('conv'), max-pooling ('pool'), and fully-connected layers ('fc'). The darker lower part of each bar shows the performance of raw predicted distances, and paler upper parts show the same after fitting a sigmoidal transform, cross-validated over both participants and stimuli. The final bars show the performance of a linearly-weighted combination of all layers. Conventions are as in Figure 4b.

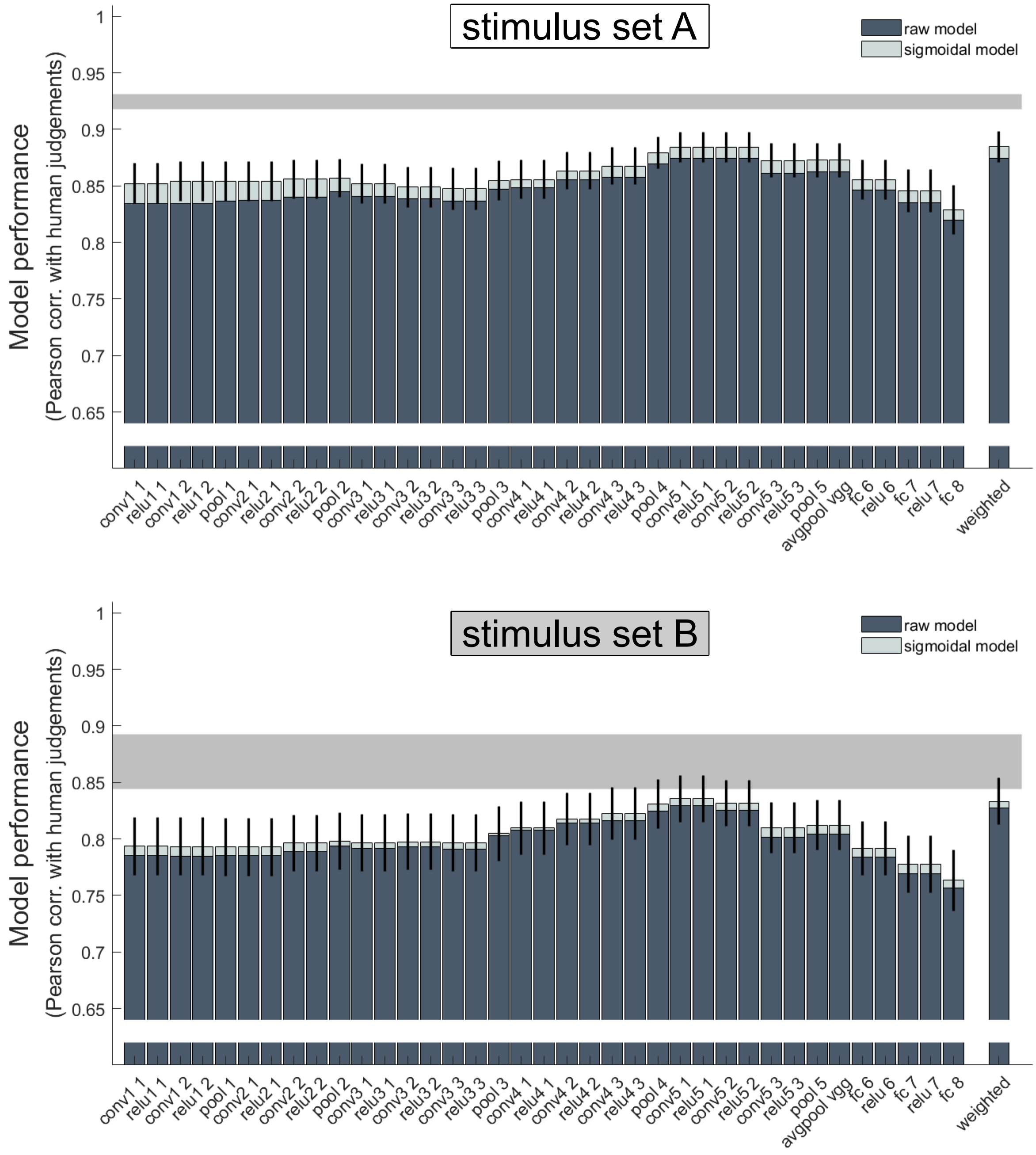

Supplementary Fig. 7. Ability of each layer within VGG-BFM-identity neural network to predict human face dissimilarity judgements.
Correlations between human dissimilarity judgements in stimulus sets A (top) and B (bottom) and Euclidean distance within each layer of the VGG-BFM-identity neural network. Conventions are as in Supplementary Figure 6.

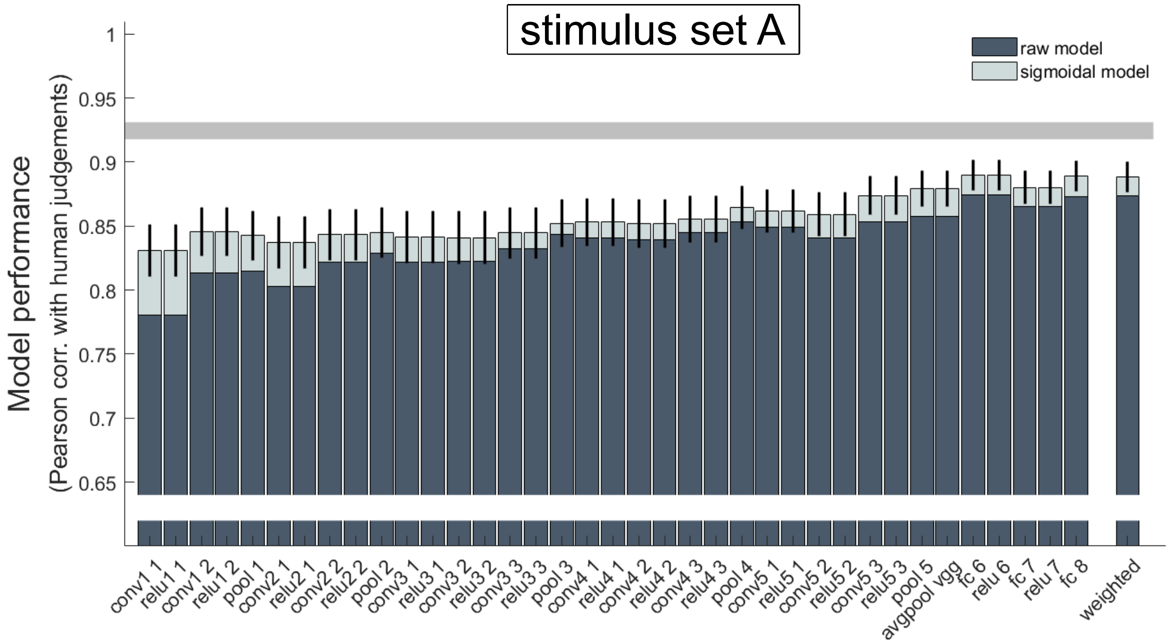

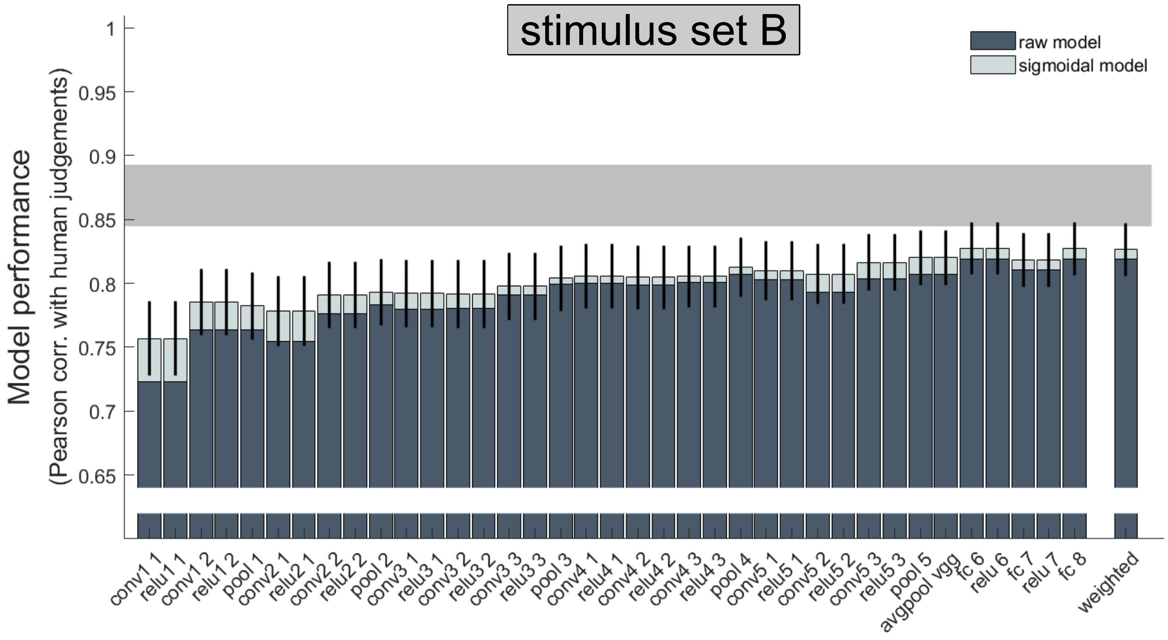

Supplementary Fig. 8. Ability of each layer within VGG-BFM-latents neural network to predict human face dissimilarity judgements.
Correlations between human dissimilarity judgements in stimulus sets A (top) and B (bottom) and Euclidean distance within each layer of the VGG-BFM-latents neural network. Conventions are as in Supplementary Figure 6.

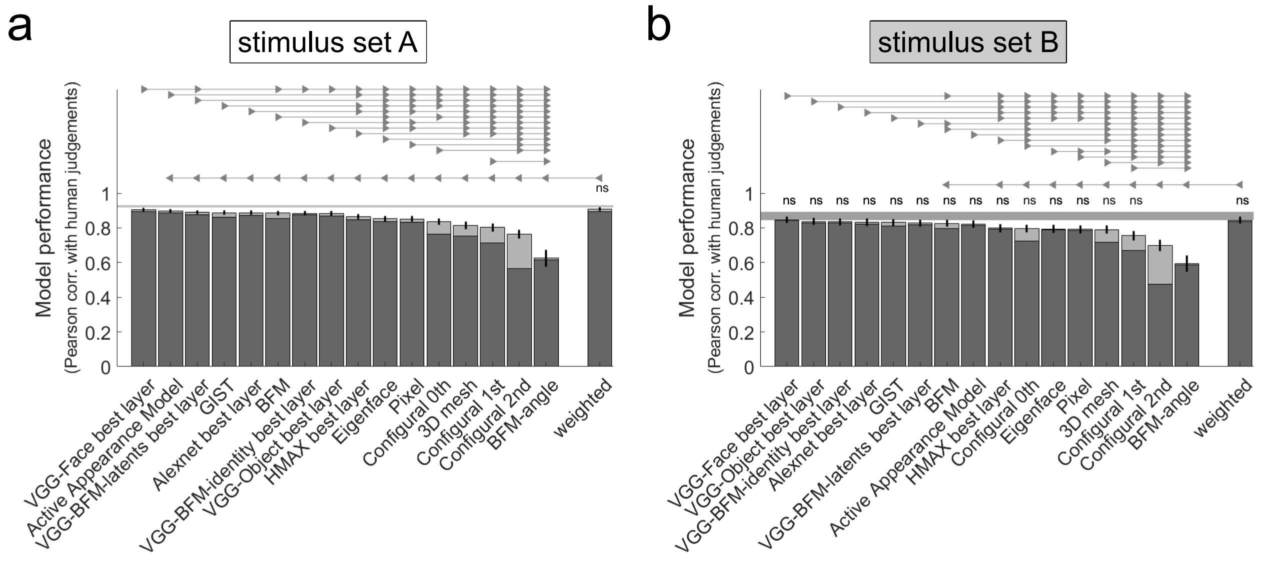

Supplementary Fig. 9. Statistical comparisons between models after allowing a sigmoidal transformation to fit human dissimilarity data.

a) Model performance data shown in Figure 4b, but with models ordered and statistically compared according to their performance after fitting a sigmoidal transform (within cross-validation folds) to raw model-predicted distances. Conventions are as in Figure 4b.

b) Corresponding data for stimulus set B.

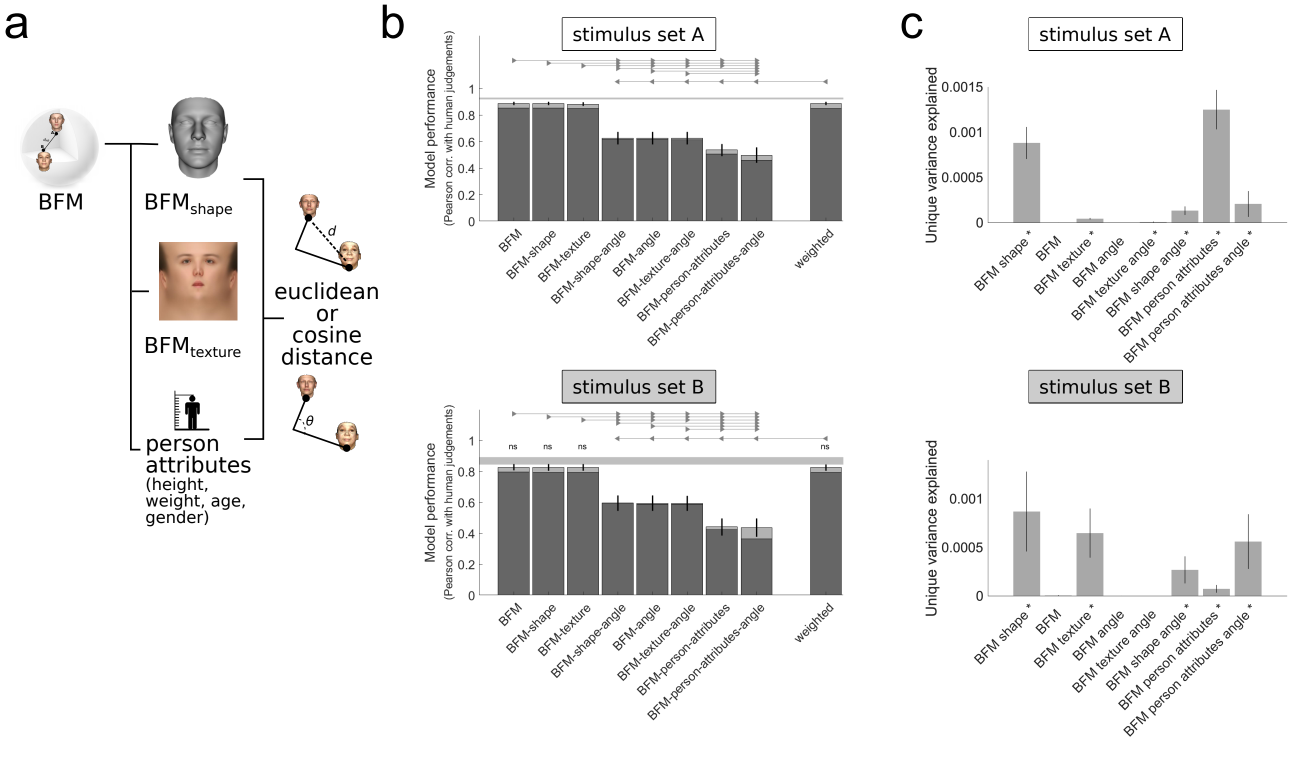

Supplementary Fig. 10. Performance of BFM models based on different subspaces and distance metrics.

a) Schematic of the six additional models presented here and their relationship to the BFM models presented in the main manuscript. Here we evaluate again the performance of a model based on either Euclidean or Cosine distance in the full 398-dimensional BFM space, as well as ones based on Euclidean or Cosine distance in: (top) the 199-dimensional shape subspace, (middle) the 199-dimensional texture subspace, or (bottom) the 4-dimensional space comprising the dimensions that capture the most variance in height, weight, age, and gender.

b) Average Pearson correlation of each model's predictions with human data in the two experimental datasets. Conventions are as in Figure 4b.

c) Unique variance analysis for the same models. Conventions are as in Figure 4c.

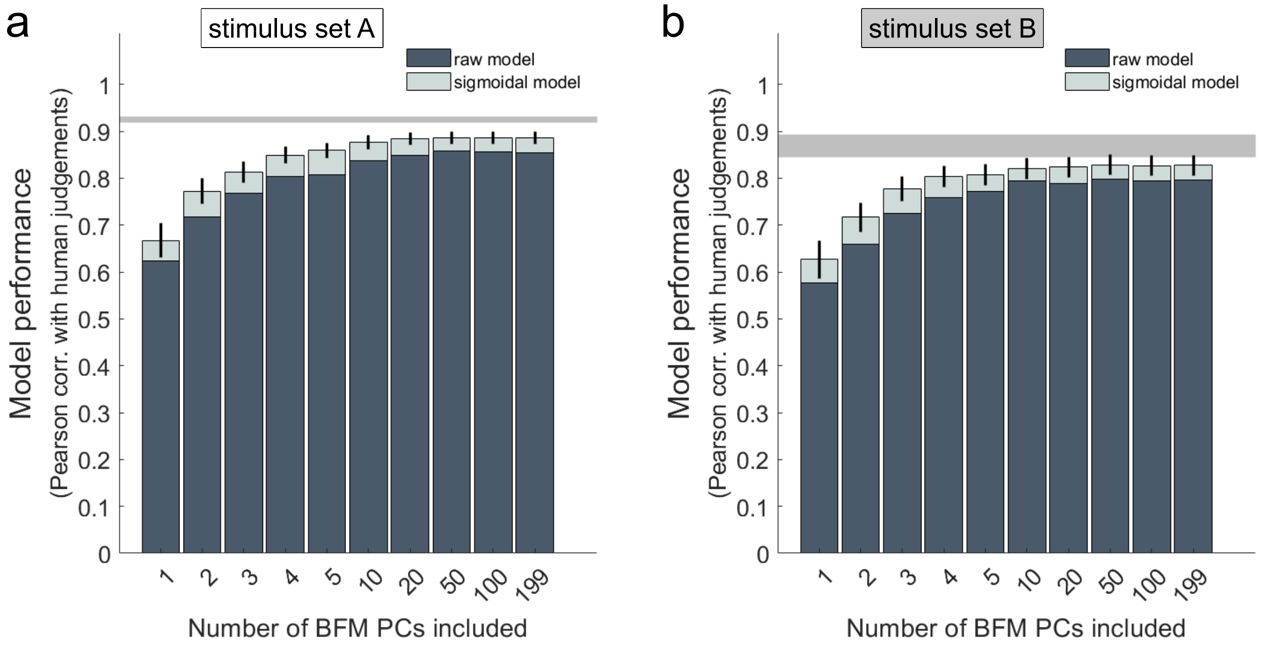

Supplementary Fig. 11. Effect of varying number of principal components in the 3D morphable Basel Face Model.

a) Correlation with human dissimilarity ratings of the stimulus set A, for models comprising the first 1, 2, 3, 4, 5, 10, 20, 50, 100, or 199 principal components from both the shape and texture sub-spaces. For example, the 1-PC model measures the Euclidean distance between faces in a two-dimensional space consisting of the first shape-PC and first texture-PC. The 199-PC model is the full BFM space included in the main manuscript analyses. Including a larger number of components aids prediction, but performance rises rapidly and saturates at a smaller dimensionality than the full BFM space.

b) Corresponding data for stimulus set B.

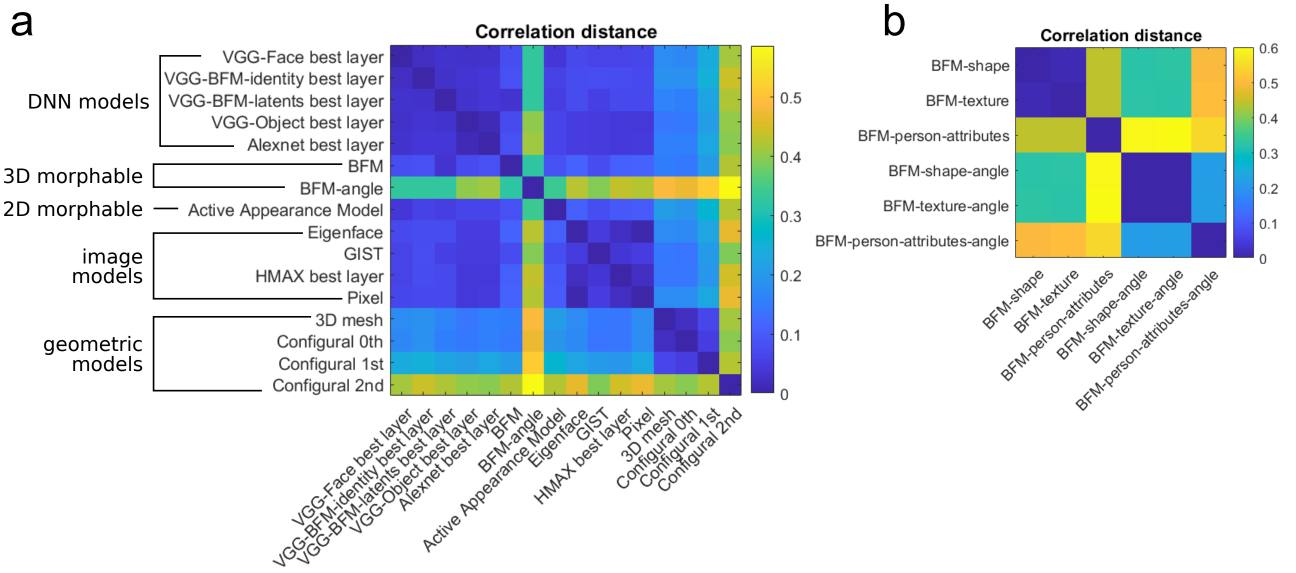

Supplementary Fig. 12. Dissimilarity (1-correlation) between model predictions for face pairs in stimulus set A.

a) Correlation distance between predictions made by models shown in the main manuscript (Figure 4), grouped by whether they derive from DNNs, from the principal components of the BFM, from simple image-computable models, or from the 3D geometry of faces.

b) Correlation distance between predictions made by models based on the BFM (see Supplementary Figure 10 for model performances).

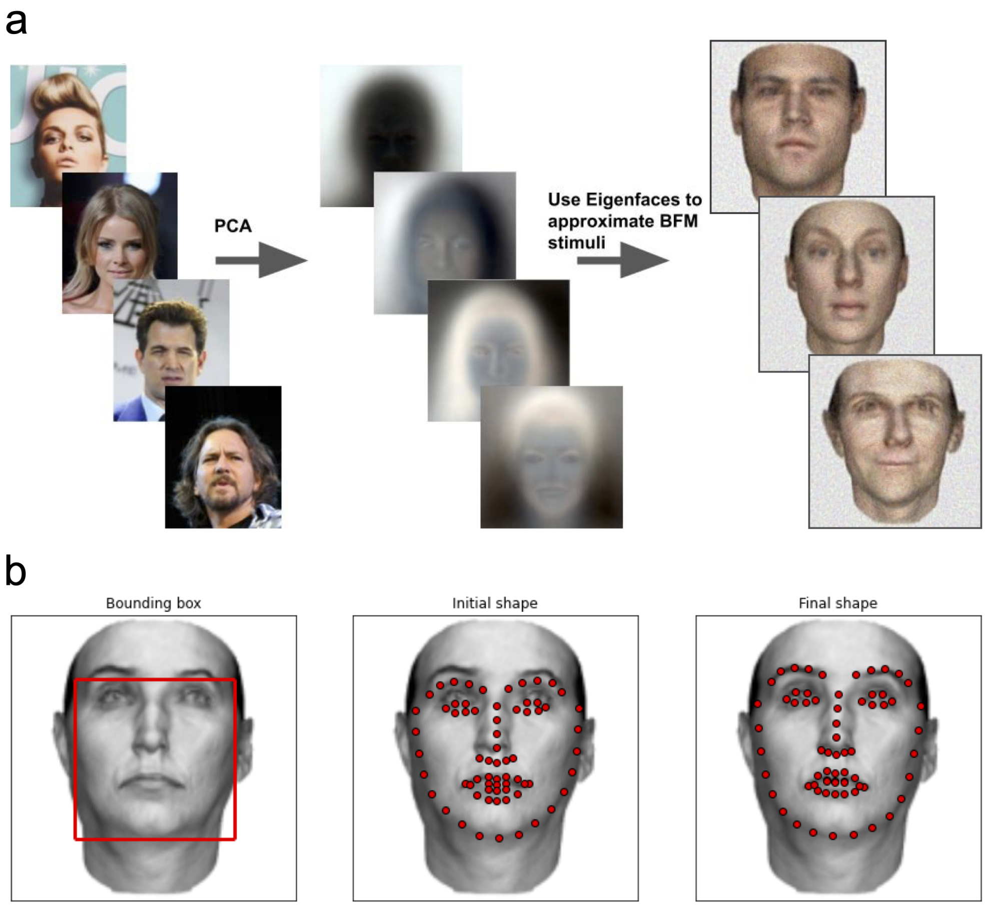

Supplementary Fig. 13. Reconstruction and fitting quality of the Eigenface and Active Appearance Models.
a) To create the Eigenface model, 4,999 eigenvectors (centre, shown reshaped to 144x144 pixels) were derived by running Principal Component Analysis on a set of 5,000 cropped and aligned real-world face photographs (left). Experimental stimulus faces were then projected into this PCA space. The Euclidean distance between the projection vectors of the two faces within each pair was taken as the model-predicted dissimilarity for that pair. The PCA space well captured the variance in stimulus images, in that stimulus faces could be well reconstructed by elementwise-multiplying their PCA projection weight vector with the 4,999 Eigenfaces (three examples shown on the right).
b) For the Active Appearance Model, we used a pretrained patch-based AAM that had been trained on a dataset of 3,283 landmark-labelled face photographs. For each stimulus face, we initialised the fitting process by providing a boundary box centred around the internal features (left), then used an iterative procedure to optimise the locations of 68 facial landmarks from their initial default positions (centre) to their final fitted positions (right).

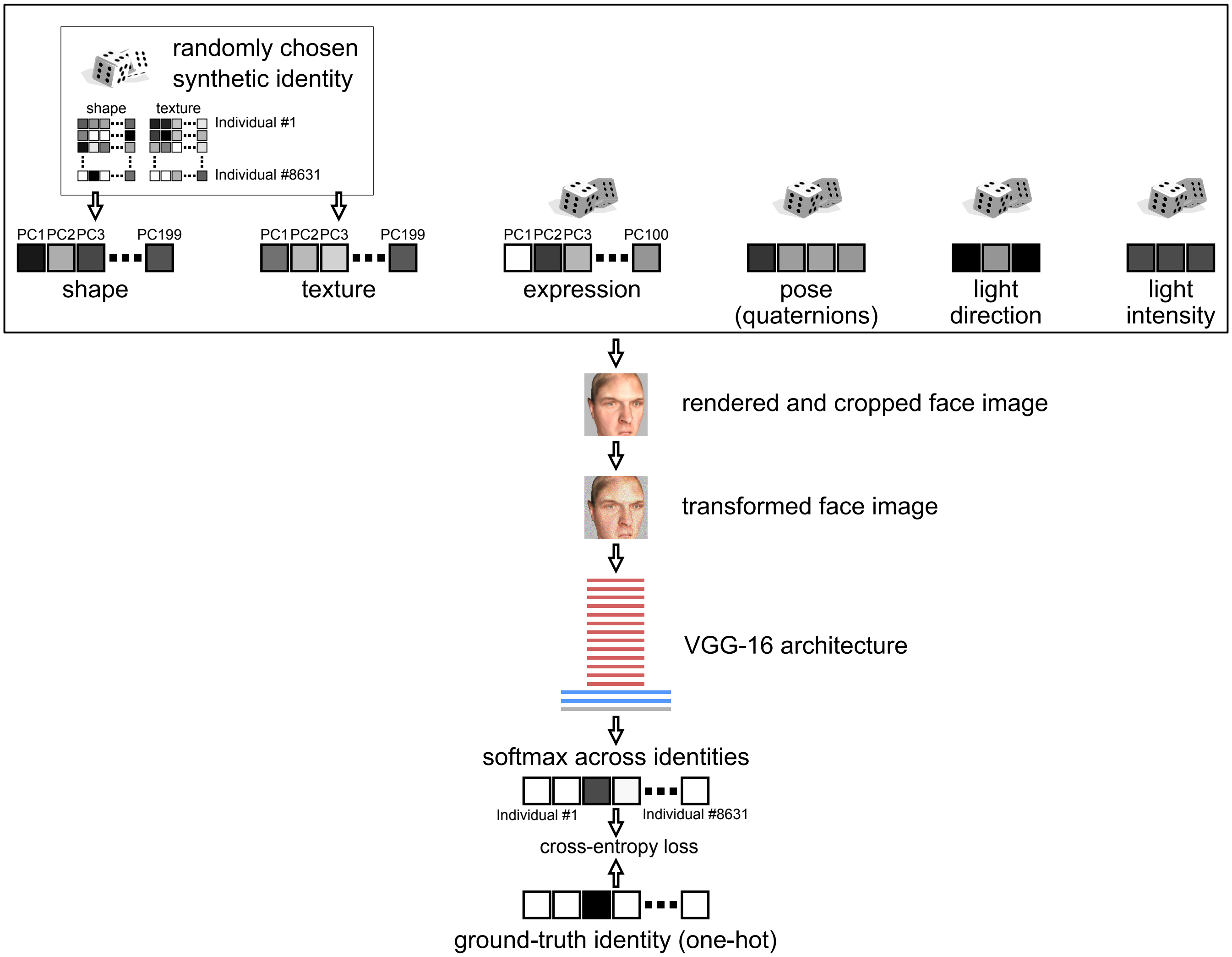

Supplementary Fig. 14. The training procedure of the VGG-BFM-identity model.
Each training or test sample pertains to one of 8,631 synthetic identities. For each synthetic identity, we sampled random shape and texture latents from the Basel Face Model distribution. The exemplars of each synthetic identity were rendered using independently sampled expression latents, pose, light direction, light intensity, and background intensity. The 3D rendering was followed by random cropping for training images or centre cropping for test images. Additional training-sample variation was introduced by 2D image transformations. The VGG-16 architecture was initialized with random weights and trained on identification, minimizing the cross-entropy loss between a 8,631-long softmax output and a one-hot representation of the ground-truth identities.

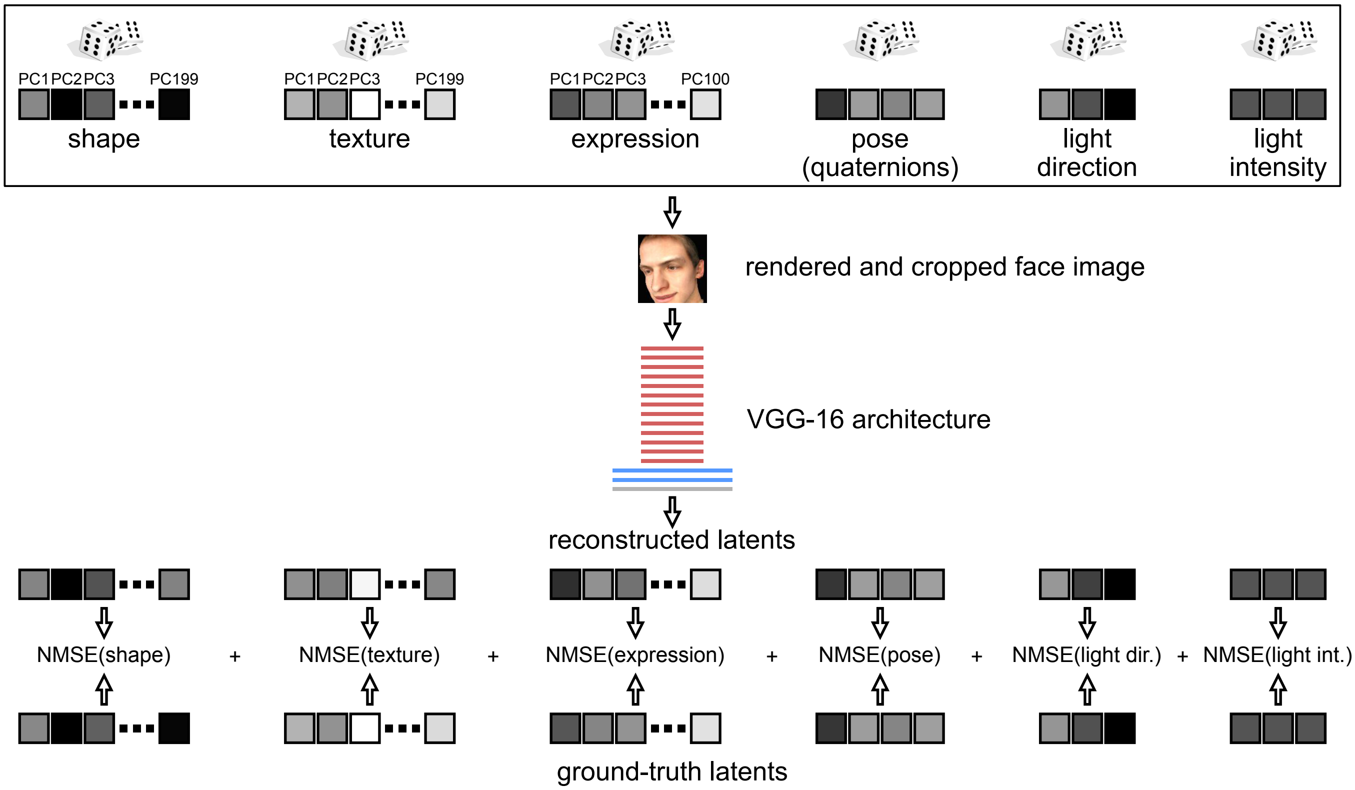

Supplementary Fig. 15. The training procedure of the VGG-BFM-latents model.
Each training or test sample was independently sampled from the Basel Face Model distribution and rendered using randomly sampled pose, light direction, light intensity, and background colour. The 3D rendering was followed by random cropping for training images or centre cropping for test images. The VGG-16 architecture was initialized with random weights and trained on recovering the latents underlying the input image, minimizing the sum of six Normalized Mean Squared Error (NMSE) terms, pertaining to BFM shape, texture, expression, pose (parameterized by quaternions), light direction, and light intensity. These error terms are computed from the squared differences between the ground-truth latents and six subsets of the 508-long output layer. Each term was normalized such that an optimal constant prediction would result in an expected NMSE of 1.0.

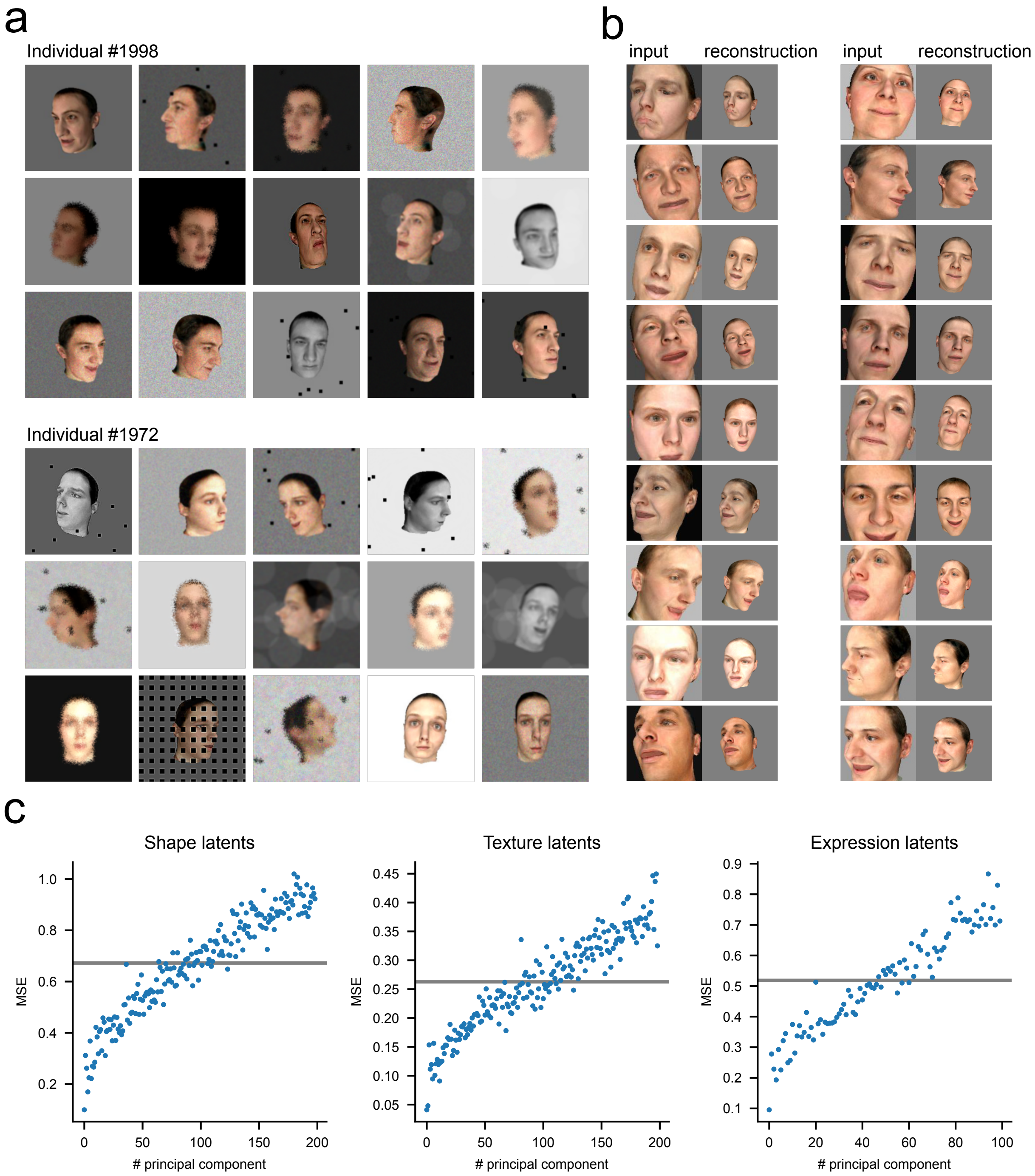

Supplementary Fig. 16. Training and validation samples, and reconstruction accuracy of the VGG-BFM-latents model.
a) A sample of VGG-BFM-identity model training set images, showing two synthetic identities and various image transformations (i.e. training data augmentation).
b) A sample of VGG-BFM-latents model validation set images along with face reconstructions using model-predicted latents.
c) Mean squared error (MSE) of each principal component in BFM latents, computed using a sample of 512 validation images. Grey lines indicate the average MSE across all principal components. The VGG-BFM-latents model successfully recovered the principal components that explain the largest variances in the BFM space, indicated by the low MSE of the first few PCs. On average across latent elements, the model reached an NMSE of 0.679 for shape, 0.266 for texture, 0.528 for expression, 0.00284 for pose, 0.00735 for light intensity, and 0.433 for light direction.
